# Impact of transgenic soybean expressing Cry1Ac and Cry1F proteins on the non-target arthropod community associated with soybean in Brazil

**DOI:** 10.1371/journal.pone.0191567

**Published:** 2018-02-02

**Authors:** Luiz H. Marques, Antonio C. Santos, Boris A. Castro, Nicholas P. Storer, Jonathan M. Babcock, Miles D. Lepping, Verissimo Sa, Valéria F. Moscardini, Dwain M. Rule, Odair A. Fernandes

**Affiliations:** 1 Dow AgroSciences Industrial Ltda, São Paulo, São Paulo, Brazil; 2 Dow AgroSciences LLC, Indianapolis, Indiana, United States of America; 3 Universidade Estadual Paulista (FCAV/UNESP), Faculdade de Ciências Agrárias e Veterinárias, FCAV/UNESP, Jaboticabal, São Paulo, Brazil; Institut Sophia Agrobiotech, FRANCE

## Abstract

Field-scale studies that examine the potential for adverse effects of *Bt* crop technology on non-target arthropods may supplement data from laboratory studies to support an environmental risk assessment. A three year field study was conducted in Brazil to evaluate potential for adverse effects of cultivating soybean event DAS-81419-2 that produces the Cry1Ac and Cry1F proteins. To do so, we examined the diversity and abundance of non-target arthropods (NTAs) in *Bt* soybean in comparison with its non-*Bt* near isoline, with and without conventional insecticide applications, in three Brazilian soybean producing regions. Non-target arthropod abundance was surveyed using Moericke traps (yellow pan) and pitfall trapping. Total abundance (N), richness (S), Shannon-Wiener (H’), Simpson’s (D) and Pielou’s evenness (J) values for arthropod samples were calculated for each treatment and sampling period (soybean growth stages). A faunistic analysis was used to select the most representative NTAs which were used to describe the NTA community structure associated with soybean, and to test for effects due to the treatments effects via application of the Principal Response Curve (PRC) method. Across all years and sites, a total of 254,054 individuals from 190 taxa were collected by Moericke traps, while 29,813 individuals from 100 taxa were collected using pitfall traps. Across sites and sampling dates, the abundance and diversity measurements of representative NTAs were not significantly affected by *Bt* soybean as compared with non-sprayed non-*Bt* soybean. Similarly, community analyses and repeated measures ANOVA, when applicable, indicated that neither *Bt* soybean nor insecticide sprays altered the structure of the NTA communities under study. These results support the conclusion that transgenic soybean event DAS-81419-2 producing Cry1Ac and Cry1F toxins does not adversely affect the NTA community associated with soybean.

## Introduction

Genetically modified (GM) crops that express Cry proteins derived from the soil bacterium, *Bacillus thuringiensis* (*Bt*), have been grown in many countries on increasing acreage since their commercial introduction in 1996 [1]. Commercially available GM crops that express *Bt* proteins are widely used to control major lepidopteran and coleopteran insect pests and are an important tool within an integrated pest management (IPM) system [2–6]. Prior to commercialization of a GM crop, a science-based environmental risk assessment (ERA) is conducted to evaluate the potential for unreasonable adverse effects on beneficial non-target organisms that occur in agricultural ecosystems [7–9]. This process has been described in detail by a number of regulatory agencies worldwide [10].

Beneficial arthropods can play an important role in regulating populations of insect pests. Thus, understanding the potential impact of pest management tools on non-target arthropod (NTA) populations in the field is an important element of ERA for transgenic crops [9]. Problem formulation is conducted in the first stage of the assessment to identify the applicable risk hypotheses and an approach to testing them, which includes estimates of hazard (effects) and exposure [11]. Knowledge about the NTA species most likely to be exposed to the proposed insecticidal protein(s) enables researchers to determine which species should be the focus of the risk assessment [12–14]. Within the scope of an ERA’s hazard assessment, a tiered testing approach is used to characterize effects of GM crops on non-target organisms at progressively higher levels of biological organization. Lower-tier tests are conducted under controlled laboratory conditions representing worst-case or artificially high exposure scenarios, while higher-tier studies are conducted under field conditions representing realistic exposure pathways and levels and natural environments [7]. For practical reasons, only a small fraction of all possible organisms can be considered for laboratory testing. To examine the potential effects of GM plants on NTAs, representative species should be selected [7, 15, 16]. The selected species should represent different ecological functions, such as herbivory, pollination, predation and parasitism of pest organisms, and decomposers [7]. The extrapolation of results from lower-tiered studies to inform risk assessments that consider the potential for field effects is well supported in the *Bt* literature, where meta-analyses examining correlation of laboratory and field data concluded that laboratory results generally predict [17] or over-estimate field effects [18]. Building on the lessons from empirical testing and the evolution of risk assessment for GM crops [7–16], the risk assessment community recognized that surrogate organism testing [14] and data transportability [19], when appropriately applied [20], provide high value through informing and simplifying risk assessments performed across geographies in which the same crop is cultivated [21]. While lower tier hazard assessments have generally predicted low risk for *Bt* crops to NTA’s under field conditions, field studies are sometimes required by government regulatory bodies allowing this prediction to be tested empirically [16].

Transgenic soybean event DAS-81419-2 (Conkesta^TM^ technology, Dow AgroSciences LLC, Indianapolis, IN) was developed via *Agrobacterium*-mediated transformation to express the Cry1Ac, Cry1F, and phosphinothricin acetyltransferase (PAT) proteins derived from *Bacillus thuringiensis* subspecies *kurstaki*, *B*. *thuringiensis* subspecies *aizawai*, and *Streptomyces viridochromogenes*, respectively. Cry1Ac and Cry1F provide protection against certain lepidopteran pests, and PAT confers tolerance to the herbicide glufosinate ammonium as a selectable marker [22]. Event DAS-81419-2 combines two *Bt* proteins in soybeans to provide South American agricultural producers with wide-spectrum protection from feeding damage by certain key lepidopteran pests [23, 24]. The Cry1Ac and Cry1F proteins expressed in event DAS-81419-2 soybean are highly similar to those expressed in cotton transformation events DAS-21023-5 and DAS-24236-5 respectively (combined through breeding in WideStrike™ cotton), which was previously assessed and approved for cultivation in the United States since 2004 [25] and in Brazil since 2009 [26]. Event DAS-81419-2 soybean has similarly been assessed and approved for cultivation in Argentina (2016) [27], Brazil (2016) [28], Canada (2014), and the United States (2014) [29].

Problem formulation conducted as part of the ERA for DAS-81419-2 soybean considered the familiarity of the mode of action for Cry proteins [30–33], the narrow spectrum of activity for Cry proteins [2, 34], the demonstrated history of safe use for *Bt* crops [35, 36] and the agronomic equivalence of DAS-81419-2 soybean to conventional soybean [37]. Previous laboratory studies using purified protein, lyophilized plant tissue, and/or leaf feeding bioassay in direct or indirect exposure test systems demonstrated no adverse effects of Cry1Ac or Cry1F on NTA [38–45]. Comprehensive reviews [2, 46] and meta-analyses [18, 47, 48] of NTA laboratory and field data have concluded the safety of transgenic *Bt* proteins across the spectrum of commercialized events and cropping systems, including cotton and maize, in which they have been deployed. The only differences in NTA populations between *Bt* crops and their conventional counterparts (in the absence of insecticide applications) have been attributed to reduction in target pest abundance or quality as prey or hosts for predators or parasitoids. Based on the existing data supporting the safety of *Bt* proteins, and Cry1Ac and Cry1F specifically, the problem formulation step for DAS-81419-2 concluded that additional testing was not required to refine the risk assessment.

Despite familiarity with *Bt* proteins, some regulatory agencies require in-country field studies to confirm the findings of lower tier studies and studies conducted in other countries. This study evaluated the potential for unreasonable adverse effects of cultivating soybean event DAS-81419-2 through a multi-year survey examining the abundance and diversity of NTAs associated with this dual *Bt* protein-expressing crop in comparison with its non-*Bt* near isoline with and without target pest management through conventional insecticide applications under field conditions. Subsequent community-level analyses were conducted with the data generated in key soybean-production regions of Brazil.

## Materials and methods

Field trials were conducted at three sites in Brazil during the 2011–2012, 2012–2013 and 2013–2014 soybean growing seasons. The selected field sites represented regions of distinct agronomic practices and environmental conditions for commercial soybean production (Table 1). Treatments included soybean event DAS-81419-2 (Conkesta^TM^ technology, Dow AgroSciences LLC, Indianapolis, IN) expressing Cry1Ac and Cry1F, a non-*Bt* near isoline without insecticides, and the same non-*Bt* variety managed under an insecticide program consistent with local commercial practices. The variety Maverick was used in all treatments [49]. Each site consisted of three replications of each treatment in a randomized complete block (RCB) design.

**Table 1:** Description of field sites used to assess the effect of *Bt* soybean event DAS-81419-2 on non-target arthropods.

Site	Latitude	Longitude	Year	Plot Size (m x m)	Planting rate (seeds/m)	Soil type
Castro, PR	24°47’34.04”S	49°53’54.94”W	2012	10 × 20	15	Loam
	24°47’34.42”S	49°54’07.23”W	2013	10 × 40	18	
Montividiu, GO	17°22’37.46”S	51°23’31.08”W	2011	9 × 20	16	Loam
	17°22’36.44”S	51°23’49.44W	2012	10 × 20	14	
	17°22’33.72”S	51°23’47.06”W	2013	10 × 40	18	
Uberlândia, MG	19°02’28.16”S	48°11’52.01”W	2011	9 × 20	15	Loam
	19°02’29.09”S	48°11’45.98”W	2012	10 × 20	10	

### Agronomic practices

All field experiments were conducted during the summer rainy season (November to January) in Brazil which paralleled commercial soybean production. All trials followed strict adherence to Brazilian regulatory requirements and were therefore conducted at accredited and certified field research stations operated by Dow AgroSciences or SGS Company. All field trials were conducted as regulated permits approved by the Comissão Técnica Nacional de Biossegurança (CTNBio). Standard agronomic practices were used for fertilization, irrigation, disease, and weed management. The tillage practices applied at each field site varied from minimum tillage (Uberlândia, MG and Montividiu, GO) to conventional tillage (Castro, PR). Thus, experiments covered the breadth of tillage practices normally observed in Brazil. Crop management practices during the study excluded the use of sprayed insecticides or miticides, except in the non-*Bt* plots managed with insecticides to simulate commercial practices. In field studies of risk assessment of *Bt* crops, the use of insecticide sprayed controls is important to reflect the replacement of existing agricultural practices [48].

### Insecticide applications

Plots managed with insecticide sprays were inspected weekly following plant emergence. At all sites, insecticide applications were performed when any of the target pest population densities exceeded economic thresholds according to local integrated pest management (IPM) programs [50], but limited to three applications, to allow comparisons across sites. The insecticide sequence used was methomyl at 107.5 g a.i./ha (Lannate® BR SL, DuPont, Wilmington, DE), followed by chlorpyrifos at 480 g a.i./ha (Lorsban® 480BR EC insecticide, Dow AgroSciences LLC, Indianapolis, IN), followed by imidacloprid + beta-cyfluthrin at 84.38 g a.i./ha (Connect® SC, Syngenta, Basel, Switzerland). This sequence was applied in all cases when the treatment threshold was met. To limit the potential for spray drift to affect untreated plots, all adjacent plots were covered with tarps during insecticide application.

### Arthropod surveys

#### Aerial arthropod sampling

Monitoring was conducted at each site using Moericke traps (yellow pan traps). These traps consisted of rectangular plastic trays (30 x 25 x 10 cm) filled with a mixture of water, formaldehyde (10%), and a few drops of liquid detergent, and were deployed during soybean growth stages V4/V5, R2, R4/R5 and R7/8 [51]. An additional early season trapping during VC/V1 was conducted for the 2012–2013 trials. At the initiation of each sampling period, two traps were placed randomly near the center of each plot and levelled at the upper third of the plant canopy. Traps were maintained for three days during each sampling period. The contents were sieved using a fine (0.5 mm) mesh sieve [52, 53], labeled and preserved with 70% ethanol, and identified in the laboratory. The most representative arthropods were identified at least to the family level; assigned to ecological function on the basis of family habits or subfamily, for families with multiple feeding habits [54].

#### Surface-dwelling arthropod sampling

Pitfall traps were used to monitor surface-dwelling arthropods during the soybean growth stages V4/V5, R2, R4/R5 and R7/8. For the 2012–2013 trials, an additional early season trapping during VC/V1 was conducted. During each sampling period, two pitfall traps were established near the center of each plot to reduce edge effects; and traps were spaced 2 m apart within a single row interspace. Each trap consisted of a plastic outer cup (12 cm in diameter x 15 cm depth) buried in the soil with the upper rim set at ground level. A galvanized tripod shield was placed over each cup with a gap of 2–3 cm between the rim of the cup and shield cover to protect against rain water and to reduce debris contamination. The traps were filled with a mixture of water, formaldehyde (10%) and a few drops of detergent, and left in the field for three days during each sampling period. Trap contents were processed as described for Aerial Arthropod Sampling.

### Statistical analyses

#### Abundance and diversity of non-target arthropods

The abundance (N), richness (S) [55], Shannon-Wiener (H’) [56], Simpson’s (D) and Pielou’s evenness (J) [57] measures for arthropod samples were calculated for each treatment and sampling time (soybean growth stage) combination, using PAST 3.12 software [58]. Diversity measures were subjected to a two-way repeated-measures analysis of variance (RM-ANOVA; α = 0.05) to test the interaction of the effect of treatments and sampling time (PROC MIXED; [59]). When the RM-ANOVA was significant for the interaction of soybean treatment with sampling time, treatment means were compared using Tukey’s test (α = 0.05). Prior to analysis, data were subjected to Shapiro-Wilk and Bartlett’s tests (α = 0.05) to check the assumptions of normal distribution and homogenous variance, respectively (PROC UNIVARIATE; [59]). Data that violated the ANOVA assumptions of normality and homogeneity of variance were log(x+1) transformed. Non-transformed means are presented.

#### Selection of representative taxa and effects on non-target arthropod communities

A faunistic analysis was used to select the most representative NTA. Faunistic analyses were performed according to Silveira Neto et al. [60] with indices calculated using ANAFAU software [61]. The most representative NTA, defined as those with the highest faunistic indices, were selected based on a combination of their dominance, abundance, frequency and consistency in the population samples. Calculations were performed for treatments within each field trial. The classification of indices was performed based on the 95% confidence interval (CI) for the mean of the total individuals collected. Before analyses, the target lepidopteran pests of *Bt* soybean were excluded. When the abundance or frequency of a taxon was higher than the upper CI limit, the taxon was classified as super-dominant (SD), super-abundant (SA), super-frequent (SF) and constant (W). If these values were within the CI limits, the taxon was classified as dominant (D), very abundant (VA) and very frequent (VF). The taxa with values below the lower CI limit were not selected by faunistic analyses and were classified as *others*. The proportion of total abundance for each taxon (number of individuals within the taxon divided by the total number of all arthropods enumerated) was also calculated.

A step-wise approach to data analysis was applied. First, the organisms selected in the faunistic analyses were used to describe components of the NTA community structure associated with soybean, and to test for effects due to the treatments by examining the Principal Response Curves (PRC). The PRC method incorporates redundancy analysis (RDA) of repeated observations via application of a direct gradient based on a linear distribution model [62]. PRC diagrams represent the part of the variation of the community structure that is explained by the first canonical axis constructed in RDA. The weight (b_k_) associated with each taxon can be interpreted as the affinity of the taxon with the Principal Response Curve (C_dt_). Only organisms with b_k_ values greater than 0.5 or lower than -0.5 are shown in the diagrams because organisms with weights between these value are likely to show either a weak response or a response that is unrelated to that depicted in the constructed curves [62]. The significance of the canonical axes produced via RDA was examined by Monte-Carlo permutation test (999 permutations; α = 0.05). Identification of a significant first axis indicates that some variation captured in the population survey is likely partly explained by the treatments, where the control treatment (non-*Bt* soybean) partly accounts for the background effect of predominant factors that influence the agroecosystem. The RDA produces estimates of the proportion of total variance explained by the constrained factors, which for this study included time (soybean growth stage) and field treatments. Prior to RDA, the abundance of organisms was log(x+1) transformed and subjected to Detrended Correspondence Analysis (DCA) to test the fit of the distribution model. The distribution model is determined by gradient length that measures the diversity in the community composition along the individual independent gradients. According to Lepš and Šmilauer [63], if the longest gradient value is larger than 4.0 the linear method would not be appropriate, while if the gradient length is less than 3.0 the linear method is a better choice. Resulting gradient lengths for the present data set were less than 3.0, indicating that application of the linear method was appropriate (S1 Table). Monte Carlo simulations, RDA and DCA were conducted using CANOCO 4.5 software [64].

When the first canonical axis was statistically significant, a two-way repeated-measures analysis of variance (RM-ANOVA; α = 0.05) was used to test the interaction of the effect of soybean treatment and sampling time for taxa that contributed most to the community response (b_k_ values greater than 0.5 or less -0.5) in the PRC diagram (PROC MIXED; [59]). RM-ANOVA was followed by Tukey’s test (α = 0.05) to separate means, when necessary.

## Results

### Non-target arthropod collections

Across all years and sites, a total of 254,054 individuals from a mean of 190 taxa were collected by Moericke traps; and 29,813 individuals from a mean of 100 taxa were collected using pitfall traps. The number of individuals collected using Moericke traps was similar among treatments, where the percentages of total individuals collected in each treatment were 33.68% non-sprayed non-*Bt* (control), 33.21% sprayed non-*Bt* and 33.11% in DAS-81419-2. The percentages of total individuals collected using pitfall traps were also similar across treatments, with 35.4, 32.5 and 32.0% in the non-sprayed non-*Bt*, sprayed non-*Bt* and DAS-81419-2 treatments, respectively (S2 and S3 Tables).

### Effects on aerial-dwelling non-target arthropods

The faunistic analysis showed a variable number of taxa selected as the most representative for each site and year (S2 Table). Via Moericke trapping, the number of taxa selected at the Castro location in 2012 and 2013 were 15 and 18 taxa, respectively. In Montividiu, the number of taxa selected by faunistic analysis were 11, 12 and 21 taxa in 2011, 2012 and 2013, respectively. In Uberlândia, the number of taxa selected were 7 and 27 in 2011 and 2012, respectively. In general, the most abundant NTAs sampled using Moericke trapping were the herbivores *Astylus variegatus* (Germar, 1824) (Coleoptera: Melyridae), *Bemisia tabaci* (Gennadius, 1889) (Hemiptera: Aleyrodidae), *Elachiptera* sp. (Diptera: Chloropidae) and the predator *Condylostylus* spp. (Diptera: Dolichopodidae).

Overall, the results of diversity analyses for aerial dwelling arthropods did not identify significant differences among treatments. However, significant differences were observed among sampling times, as soybean crop phenology progressed (Table 2). The abundance (N) of NTAs was higher in DAS-81419-2 plots than in the non-sprayed non-*Bt* and sprayed non-*Bt* at Castro 2012 mainly during reproductive stages (S4 Table) whereas richness (S) associated with DAS-81419-2 was similar to the control at the same location in 2013. Arthropod diversity associated with DAS-81419-2, measured by Shannon´s index (H’) was similar to the non-sprayed non-*Bt* and significantly higher than the sprayed non-*Bt* in Uberlândia 2011 (Table 2 and S4 Table).

**Table 2:** Two-way repeated-measures ANOVA results for abundance (N), richness (S), Shannon’s diversity index (H’), Simpson’s diversity index (D) and Pielou’s evenness index (J) of non-target arthropods collected by Moericke traps (yellow pan) in non-*Bt* (with and without insecticides) and *Bt* (DAS-81419-2) soybean fields at three sites over two to three years in Brazil.

Site	Year	Diversity indices	Soybean treatments			Two-way ANOVA					
			Non-sprayed non-*Bt*	Sprayed non-*Bt*	DAS-81419-2	Soybean treat. (A)		Sampling time (B)		Interaction (A x B)	
						*F*	*P*	*F*	*P*	*F*	*P*
Castro	2012	N	1161.50 ± 122.52 b	966.25 ± 126.53 c	1418.25 ± 165.52 a	39.78	**0.002**	155.06	**< 0.001**	8.58	**< 0.001**
		S	28.08 ± 1.47	26.83 ± 1.27	32.42 ± 1.40	5.29	0.075	5.93	**0.032**	0.83	0.572
		H’	1.86 ± 0.05	1.89 ± 0.08	1.83 ± 0.10	0.76	0.525	481.13	**< 0.001**	7.49	**0.002**
		D	0.77 ± 0.01	0.78 ± 0.02	0.74 ± 0.02	2.66	0.184	129.84	**< 0.001**	8.35	**0.001**
		J	0.24 ± 0.02 ab	0.26 ± 0.02 a	0.20 ± 0.02 b	13.76	**0.016**	36.89	**< 0.001**	3.56	**0.029**
	2013	N	220.60 ± 25.07	208.60 ± 15.73	204.00 ± 22.47	0.61	0.588	6.38	**0.013**	0.23	0.980
		S	29.40 ± 1.77 ab	31.33 ± 1.78 a	25.93 ± 1.50 b	8.85	**0.034**	14.14	**0.001**	0.97	0.491
		H’	2.40 ± 0.05	2.49 ± 0.10	2.31 ± 0.06	4.34	0.099	7.91	**0.007**	2.03	0.109
		D	0.83 ± 0.01	0.84 ± 0.02	0.83 ± 0.01	0.55	0.614	6.62	**0.012**	1.78	0.156
		J	0.41 ± 0.04	0.41 ± 0.02	0.42 ± 0.03	0.02	0.979	5.18	**0.023**	0.23	0.980
Montividiu	2011	N	330.42 ± 76.83	368.58 ± 77.27	324.92 ± 61.71	1.59	0.311	84.23	**< 0.001**	4.55	**0.012**
		S	24.17 ± 1.35	24.17 ± 1.29	25.25 ± 1.22	0.44	0.674	18.17	**0.002**	0.58	0.737
		H’	1.74 ± 0.17	1.62 ± 0.10	1.71 ± 0.11	2.07	0.242	21.57	**0.001**	2.59	0.076
		D	0.67 ± 0.05	0.64 ± 0.03	0.64 ± 0.03	0.77	0.523	10.91	**0.008**	2.73	0.066
		J	0.26 ± 0.03	0.22 ± 0.02	0.23 ± 0.02	1.65	0.299	17.85	**0.002**	3.20	**0.041**
	2012	N	596.50 ± 227.99	581.75 ± 219.17	896.17 ± 441.55	0.35	0.723	83.34	**< 0.001**	2.34	0.099
		S	24.67 ± 2.57	24.25 ± 3.04	20.25 ± 2.35	4.94	0.083	10.72	**0.008**	1.64	0.219
		H’	1.67 ± 0.13	1.68 ± 0.14	1.59 ± 0.17	1.98	0.252	64.04	**< 0.001**	0.73	0.637
		D	0.67 ± 0.04	0.69 ± 0.05	0.65 ± 0.05	1.28	0.372	40.45	**< 0.001**	1.19	0.372
		J	0.26 ± 0.05	0.28 ± 0.05	0.32 ± 0.06	1.75	0.285	70.43	**< 0.001**	1.95	0.153
	2013	N	395.93 ± 81.60	409.87 ± 85.60	460.87 ± 85.34	0.96	0.458	43.43	**< 0.001**	0.92	0.524
		S	28.80 ± 2.53	29.27 ± 2.60	31.73 ± 2.32	4.86	0.085	60.57	**< 0.001**	4.89	**0.003**
		H’	1.72 ± 0.10	1.63 ± 0.15	1.62 ± 0.13	0.65	0.570	16.04	**< 0.001**	1.50	0.233
		D	0.63 ± 0.04	0.58 ± 0.05	0.57 ± 0.04	1.29	0.370	17.20	**< 0.001**	1.75	0.161
		J	0.23 ± 0.04	0.21 ± 0.04	0.19 ± 0.04	0.98	0.448	43.77	**< 0.001**	1.62	0.197
Uberlândia	2011	N	353.00 ± 53.59	390.42 ± 69.60	376.00 ± 61.37	0.20	0.826	29.37	**< 0.001**	5.18	**0.008**
		S	39.17 ± 2.38	39.33 ± 2.87	45.08 ± 4.77	2.94	0.164	21.59	**0.001**	5.91	**0.004**
		H’	2.34 ± 0.06 ab	2.33 ± 0.13 b	2.51 ± 0.05 a	8.99	**0.033**	16.64	**0.003**	3.23	**0.040**
		D	0.80 ± 0.02	0.79 ± 0.02	0.83 ± 0.01	4.94	0.083	19.79	**0.002**	3.66	**0.026**
		J	0.29 ± 0.03	0.29 ± 0.04	0.31 ± 0.03	0.59	0.594	29.71	**< 0.001**	9.38	**< 0.001**
	2012	N	3918.50 ± 1045.77	3951.08 ± 858.45	3163.08 ± 950.32	0.99	0.446	39.60	**< 0.001**	5.76	**0.005**
		S	60.92 ± 3.50	54.42 ± 5.06	61.83 ± 4.42	0.77	0.520	68.16	**< 0.001**	2.36	0.096
		H’	1.33 ± 0.18	1.33 ± 0.13	1.62 ± 0.20	5.35	0.074	58.33	**< 0.001**	3.38	**0.035**
		D	0.50 ± 0.07	0.56 ± 0.05	0.58 ± 0.07	6.43	0.056	53.29	**< 0.001**	1.82	0.178
		J	0.08 ± 0.02	0.10 ± 0.03	0.10 ± 0.02	5.39	0.073	59.40	**< 0.001**	5.93	**0.004**

*P*-values highlighted in bold are statistically significant (α = 0.05).

Degrees of freedom: soybean treatments = 2; sampling time = 3 (2011 and 2012) and 4 (2013); interaction = 6 (2011 and 2012) and 8 (2013); residual = 12 (2011 and 2012) and 16 (2013).

Means (± SE) followed by different letters were significantly different within rows (Tukey’s test, α = 0.05).

Abundance was log(x + 1) transformed prior to analysis. Non-transformed means are presented.

**Fig 1:**
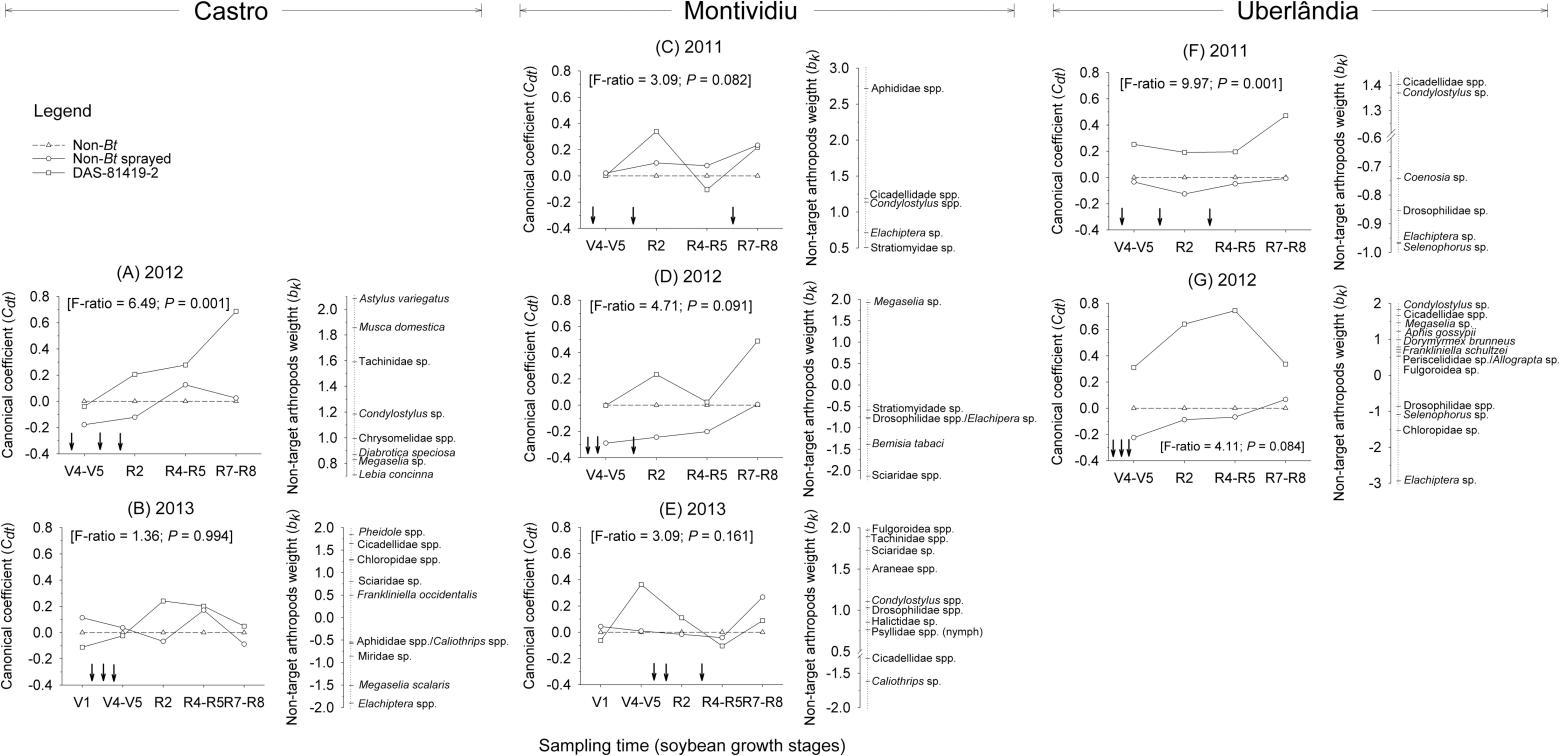
Principal Response Curves (PRC) indicating the effect of non-*Bt* soybean with insecticides sprayed and *Bt* soybean (DAS-81419-2) on the most representative non-target arthropods collected by Moericke traps (yellow pan) at three sites over two to three years in Brazil. Dotted line indicates the community response to the control treatment (non-*Bt* without insecticides). The non-target arthropods weight (*b*_*k*_) can be interpreted as the affinity of the taxon with the Principal Response Curves (*C*_*dt*_). Only taxa with *b*_*k*_ values greater than 0.5 and less than -0.5 are shown in the diagram. Following Monte Carlo permutation tests, *P*-values less than 0.05 indicate a statistically significant difference in community response between at least one of the treatments and the control. Down arrows (↓) indicate the time of insecticide application in the sprayed non-*Bt* treatment.

The redundancy analysis (RDA) results for the most representative NTAs collected by Moericke trapping in Castro (2012) was significant for the first canonical axis (F = 6.49; P = 0.001) (S5 Table, Fig 1A). The first axis explained 37.5% of the variation of the aerial NTA community sampled. Of all the variance, 56.3% was attributed to the sampling time and 8.8% to the soybean treatments (S5 Table). Abundance of several taxa was higher in DAS-81419-2 plots compared with the non-sprayed non-*Bt* and sprayed non-*Bt* treatments as crop phenology progressed (Fig 1A). In 2013, no significant patterns were observed across treatments at Castro (first axis: F = 1.36; P = 0.994) (S5 Table, Fig 1B).

In Montividiu during 2011 and 2012 the first axis of RDA analysis was not significant (2011: F = 3.09; P = 0.082; 2012: F = 4.71; P = 0.091) (S5 Table, Fig 1C and 1D). The Principal Response Curve (PRC) analysis identified changes in the aerial NTA community structure where 67.3% of the variation was explained by sampling time and 5.8% by soybean treatment (S5 Table). The sprayed non-*Bt* and DAS-81419-2 treatments presented different canonical coefficients (C_dt_) over sampling periods, where taxa in the sprayed non-*Bt* treatment showed a negative response and the taxa response in the DAS-81419-2 treatment was positive compared with the non-sprayed non-*Bt* treatment. In 2013, the first axis (F = 3.09; P = 0.161) was not significant (S5 Table, Fig 1E).

In Uberlândia, during 2011, the RDA results indicated a significant first axis (F = 9.97; P = 0.001) (S5 Table, Fig 1F). The first axis explained 62.3% of the variation of the aerial NTA community structure, wherein 59.3% was explained by sampling time and 10.9% by soybean treatment (S5 Table). In this case, the PRC response for DAS-81419-2 was positive over the sampling time whereas the sprayed non-*Bt* was negative compared with the non-sprayed non-*Bt* treatment. The NTAs that followed the observed trends were Cicadellidae spp. and *Condylostylus* sp., whereas *Coenosia* sp. (Diptera: Muscidae), Drosophilidae sp. (Diptera), *Elachiptera* sp. and *Selenophorus* sp. showed negative weights and responded oppositely to the trends (Fig 1F). In 2012, the first axis was not significant (F = 4.11; P = 0.084) (S5 Table, Fig 1G). We observed higher abundance for most of NTA in the DAS-81419-2 plots except for some Diptera (*Elachiptera* sp., Chloropidae sp., Drosophilidae spp.), and the carabid beetle, *Selenophorus* sp. (Fig 1G).

**Fig 2:**
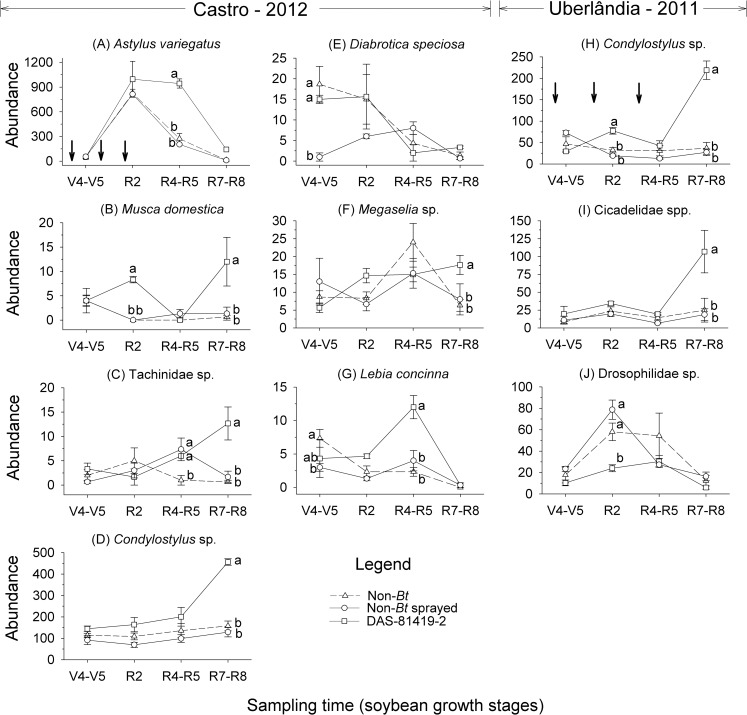
Population dynamics for non-target arthropod groups collected by Moericke traps (yellow pan) in non-*Bt* (with and without insecticides) and *Bt* (DAS-81419-2) soybean fields. Means (± SE) within sampling time followed by different letters are significantly different (Tukey’s test, α = 0.05). Taxa shown include those with taxon weights greater than 0.5 and less -0.5 which were associated with statistically significant Monte Carlo tests. Down arrows (↓) indicate the time of insecticide application in the sprayed non-*Bt* treatment.

The first axis was significant only for the field trials at Castro in 2012 and Uberlândia in 2011. Applying the step-wise data analysis for these trials, we investigated the effect of soybean treatments and sampling times (two-way RM-ANOVA) for NTA that contributed the most to the community response (weights greater than 0.5 or less -0.5). For Castro in 2012, the abundance of *A*. *variegatus* was higher in DAS-81419-2 plots compared with the other treatments. The abundance of *Condylostylus* sp. and *Lebia concinna* Brullé, 1838 (Coleoptera: Carabidae) were also higher in the DAS-81419-2 treatment compared with the sprayed non-*Bt* treatment. Only the abundances of *Musca domestica* (Linnaeus, 1758) (Diptera: Muscidae), and other Diptera (Tachinidae sp., *Megaselia* sp.) were not influenced by sampling time (Table 3). Significant interactions were observed for soybean treatment and sampling time for *A*. *variegatus*, *M*. *domestica*, Tachinidae sp., *Condylostylus* sp., *Diabrotica speciosa* (Germar, 1824) (Coleoptera: Chrysomelidae), *Megaselia* sp. and *L*. *concinna* (Table 3, Fig 2A–2G).

**Table 3:** Two-way repeated-measures ANOVA results for abundance of non-target arthropods with values weights greater than 0.5 and less -0.5 in the Principal Response Curves (PRC) analysis with first axis significant collected by Moericke traps (yellow pan) in non-*Bt* (with and without insecticides) and *Bt* (DAS-81419-2) soybean fields at two sites over two to three years in Brazil.

Site (year)	Non-target arthropods	Soybean treatments			Two-way ANOVA					
		Non-sprayed non-*Bt*	Sprayed non-*Bt*	DAS-81419-2	Soybean treat. (A)		Sampling time (B)		Interaction (A x B)	
					*F*	*P*	*F*	*P*	*F*	*P*
Castro (2012)	*Astylus variegatus*	290.00 ± 99.45 b	270.58 ± 97.21 b	532.67 ± 140.85 a	12.58	**0.019**	114.25	**< 0.001**	7.58	**0.002**
	*Musca domestica*	1.17 ± 0.55	1.67 ± 0.61	6.08 ± 1.82	6.97	0.050	2.84	0.128	5.21	**0.007**
	Tachinidae sp.	2.17 ± 0.83	3.17 ± 1.01	5.92 ± 1.54	6.17	0.060	1.99	0.217	4.81	**0.010**
	*Condylostylus* sp.	128.92 ± 11.15 ab	97.17 ± 10.18 b	241.33 ± 39.95 a	10.57	**0.025**	45.83	**< 0.001**	20.28	**< 0.001**
	Chrysomelidae spp.	23.67 ± 6.16	14.92 ± 3.50	28.41 ± 7.19	5.54	0.070	65.00	**< 0.001**	2.78	0.062
	*Diabrotica speciosa*	9.83 ± 2.73	3.92 ± 1.04	9.00 ± 2.60	3.43	0.135	4.93	**0.047**	4.15	**0.017**
	*Megaselia* sp.	11.83 ± 2.50	10.75 ± 2.22	13.25 ± 1.63	0.26	0.780	3.89	0.074	3.95	**0.020**
	*Lebia concinna*	3.00 ± 0.89 ab	2.17 ± 0.56 b	5.33 ± 1.46 a	7.92	**0.041**	13.48	**0.004**	5.63	**0.005**
Uberlândia (2011)	*Condylostylus* sp.	36.67 ± 5.82 b	33.17 ± 7.41 b	92.33 ± 23.36 a	27.02	**0.005**	96.81	**< 0.001**	18.37	**< 0.001**
	Cicadellidae spp.	17.83 ± 4.41 b	14.00 ± 2.79 b	45.08 ± 12.88 a	26.05	**0.005**	3.93	0.073	5.95	**0.004**
	*Coenosia* sp.	14.58 ± 2.79	13.33 ± 2.72	9.58 ± 2.57	3.79	0.119	12.77	**0.005**	2.24	0.111
	Drosophilidae sp.	35.58 ± 8.13	36.42 ± 7.77	17.67 ± 3.35	4.69	0.089	19.63	**0.002**	3.48	**0.031**
	*Selenophorus* sp.	18.50 ± 7.57	49.58 ± 17.24	26.83 ± 18.46	0.89	0.477	4.08	0.067	0.92	0.516
	*Elachiptera* sp.	144.17 ± 32.61 a	153.42 ± 28.69 a	72.58 ± 18.69 b	53.59	**0.001**	47.01	**< 0.001**	2.08	0.132

*P*-values highlighted in bold are statistically significant (α = 0.05).

Degrees of freedom: soybean fields = 2; sampling time = 3; interaction = 6; residual = 12.

Means (± SE) followed by different letters were significantly different within rows (Tukey’s test, α = 0.05).

In Uberlândia in 2011, the abundances of *Condylostylus* sp. and Cicadellidae spp. (Hemiptera) were higher in the DAS-81419-2 treatment, whereas the abundance of *Elachiptera* sp. was lower compared to the other treatments. In this trial, only the abundances of Cicadellidae spp. and *Selenophorus* sp. were not influenced by sampling time (Table 3). Significant interactions between soybean treatment and sampling time were observed for *Condylostylus* sp., Cicadellidae spp. and Drosophilidae sp. (Table 3, Fig 2H–2J), where abundances in DAS-81419-2 plots were also generally similar to or greater than those of non-*Bt* soybean.

### Effects on surface-dwelling non-target arthropods

Via pitfall trapping in the Castro trials, the number of representative taxa selected by faunistic analysis was 10 in 2012 and 20 in 2013 (S3 Table). In Montividiu, the number of taxa selected was 10 in 2011 and 2012, and 6 in 2013. In the locality of Uberlândia, the number of taxa selected was 10 in 2011 and 16 in 2012. In general, the most abundant surface-dwelling NTAs collected by pitfall traps were detritivores (Collembola), omnivores (*Pheidole* spp. [Hymenoptera: Formicidae] and *Selenophorus* sp. [Coleoptera: Carabidae]), and herbivores (*B*. *tabaci*).

Similar to the results from the aerial dwelling arthropod survey, diversity analyses for surface dwelling arthropods did not identify significant differences among treatments; but significant differences were observed among sampling times (Table 4). Shannon’s diversity index (H’), Simpson’s diversity index (D), and Pielou’s evenness index (J) measures for surface-dwelling arthropods were higher for the DAS-81419-2 treatment than the non-sprayed non-*Bt* treatment at Castro in 2012 and Montividiu in 2011. For other measures, abundance (N) and richness (S) were lower in the DAS-81419-2 treatment than the non-sprayed non-*Bt* treatment for Montividiu in 2012 (Table 4). In addition, the abundance and diversity of surface-dwelling arthropods varied with sampling dates, but in few cases was there an interaction between sampling dates and soybean treatments (S6 Table). Simpson’s diversity index (D) values for arthropods in these cases were higher in the DAS-81419-2 treatment than in the non-sprayed non-*Bt* treatment, mainly during the reproductive stages R4-R5 and R7-R8 at Castro in 2012. Similarly, DAS-81419-2 plots were associated with higher richness (S) than non-sprayed non-*Bt* plots during the reproductive stages R2 and R4-R5 at Uberlândia in 2011 (S6 Table). Thus, considering all growing seasons (years) and sites, the community of surface-dwelling insects was similar across treatments and, in some cases, DAS-81419-2 supported a higher abundance and diversity than the non-*Bt* treatments (Table 4 and S6 Table).

**Table 4:** Two-way repeated-measures ANOVA results for abundance (N), richness (S), Shannon’s diversity index (H’), Simpson’s diversity index (D) and Pielou’s evenness index (J) of non-target arthropods collected by pitfall traps in non-*Bt* (with and without insecticides) and *Bt* (DAS-81419-2) soybean fields at three sites over two to three years in Brazil.

Site	Year	Diversity indices	Soybean treatments			Two-way ANOVA					
			Non-sprayed non-*Bt*	Sprayed non-*Bt*	DAS-81419-2	Soybean treat. (A)		Sampling time (B)		Interaction (A x B)	
						*F*	*P*	*F*	*P*	*F*	*P*
Castro	2012	N	74.75 ± 15.05	64.83 ± 10.59	61.17 ± 9.91	0.23	0.809	87.09	**< 0.001**	0.88	0.537
		S	11.33 ± 1.14	12.67 ± 0.92	14.00 ± 1.50	4.41	0.097	5.57	**0.036**	0.37	0.884
		H’	1.63 ± 0.10 b	1.90 ± 0.07 a	2.00 ± 0.09 a	34.27	**0.003**	3.42	0.093	2.23	0.112
		D	0.71 ± 0.03 b	0.77 ± 0.02 a	0.80 ± 0.02 a	18.70	**0.009**	8.18	**0.015**	4.89	**0.009**
		J	0.52 ± 0.06	0.55 ± 0.02	0.57 ± 0.03	0.71	0.544	15.23	**0.003**	3.39	**0.034**
	2013	N	63.47 ± 16.17	49.27 ± 12.77	51.80 ± 9.88	0.54	0.616	18.69	**< 0.001**	0.33	0.945
		S	13.93 ± 1.42	12.93 ± 1.07	12.13 ± 1.09	1.03	0.440	4.12	**0.042**	0.37	0.924
		H’	1.91 ± 0.12	2.05 ± 0.12	1.80 ± 0.08	1.39	0.346	2.73	0.106	1.38	0.276
		D	0.75 ± 0.04	0.81 ± 0.03	0.74 ± 0.03	0.60	0.594	3.53	0.061	1.37	0.282
		J	0.59 ± 0.06	0.69 ± 0.06	0.59 ± 0.06	0.76	0.525	13.82	**0.001**	0.79	0.616
Montividiu	2011	N	33.08 ± 4.57	25.92 ± 5.96	21.25 ± 4.61	3.13	0.152	4.14	0.066	0.74	0.627
		S	9.92 ± 0.76	10.17 ± 0.98	7.83 ± 1.21	4.27	0.102	3.57	0.086	0.73	0.637
		H’	1.83 ± 0.09	1.97 ± 0.12	1.69 ± 0.16	3.61	0.127	1.38	0.337	1.88	0.166
		D	0.77 ± 0.03	0.81 ± 0.03	0.75 ± 0.05	2.31	0.215	0.99	0.459	2.60	0.075
		J	0.66 ± 0.05 b	0.76 ± 0.06 a	0.78 ± 0.04 a	17.61	**0.010**	4.15	0.065	1.96	0.151
	2012	N	107.83 ± 45.82 a	84.58 ± 18.29 ab	41.58 ± 7.85 b	8.91	**0.034**	12.34	**0.006**	0.36	0.891
		S	17.25 ± 1.39 a	16.50 ± 1.54 a	11.75 ± 1.17 b	17.55	**0.010**	22.43	**0.001**	0.75	0.621
		H’	2.21 ± 0.15	2.11 ± 0.09	1.93 ± 0.13	2.08	0.241	8.59	**0.014**	1.11	0.412
		D	0.82 ± 0.03	0.81 ± 0.02	0.79 ± 0.02	0.36	0.719	4.54	0.055	0.74	0.625
		J	0.58 ± 0.16	0.54 ± 0.06	0.63 ± 0.05	1.18	0.395	11.02	**0.007**	0.10	0.995
	2013	N	17.20 ± 3.96	18.33 ± 4.51	15.20 ± 4.20	0.57	0.603	8.11	**0.006**	0.53	0.813
		S	7.87 ± 1.06	7.67 ± 0.89	7.27 ± 0.85	0.22	0.812	3.38	0.067	0.47	0.863
		H’	1.71 ± 0.15	1.71 ± 0.13	1.67 ± 0.10	0.14	0.871	4.35	**0.037**	0.451	0.873
		D	0.75 ± 0.04	0.76 ± 0.03	0.75 ± 0.03	0.21	0.818	5.83	**0.017**	0.45	0.872
		J	0.80 ± 0.07	0.79 ± 0.05	0.80 ± 0.06	0.04	0.965	9.55	**0.004**	0.36	0.927
Uberlândia	2011	N	87.25 ± 11.06	112.33 ± 28.21	113.50 ± 18.97	0.69	0.551	15.17	**0.003**	1.02	0.458
		S	17.25 ± 1.14	18.75 ± 1.40	19.83 ± 1.51	4.30	0.101	1.86	0.238	6.74	**0.003**
		H’	2.18 ± 0.09	2.24 ± 0.05	2.17 ± 0.13	0.37	0.712	0.03	0.994	2.47	0.086
		D	0.82 ± 0.02	0.84 ± 0.01	0.80 ± 0.03	1.44	0.339	0.03	0.993	0.96	0.492
		J	0.53 ± 0.03	0.53 ± 0.04	0.48 ± 0.05	0.72	0.543	2.35	0.172	0.691	0.662
	2012	N	475.75 ± 79.51	436.50 ± 73.25	475.00 ± 125.25	0.48	0.650	67.13	**< 0.001**	1.76	0.191
		S	21.58 ± 1.36	23.67 ± 1.78	23.58 ± 2.06	1.51	0.325	1.59	0.286	1.68	0.208
		H’	1.44 ± 0.16 b	1.86 ± 0.12 a	1.60 ± 0.15 ab	12.79	**0.018**	27.48	**< 0.001**	2.43	0.090
		D	0.57 ± 0.06 b	0.71 ± 0.04 a	0.61 ± 0.06 ab	8.24	**0.038**	34.97	**< 0.001**	2.84	0.059
		J	0.22 ± 0.03	0.30 ± 0.03	0.25 ± 0.04	3.88	0.116	16.14	**0.003**	1.62	0.225

*P*-values highlighted in bold are statistically significant (α = 0.05).

Degrees of freedom: soybean treatments = 2; sampling time = 3 (2011 and 2012) and 4 (2013); interaction = 6 (2011 and 2012) and 8 (2013); residual = 12 (2011 and 2012) and 16 (2013).

Means (± SE) followed by different letters were significantly different within rows (Tukey’s test, α = 0.05).

Abundance was log(x + 1) transformed prior to analysis. Non-transformed means are presented.

**Fig 3:**
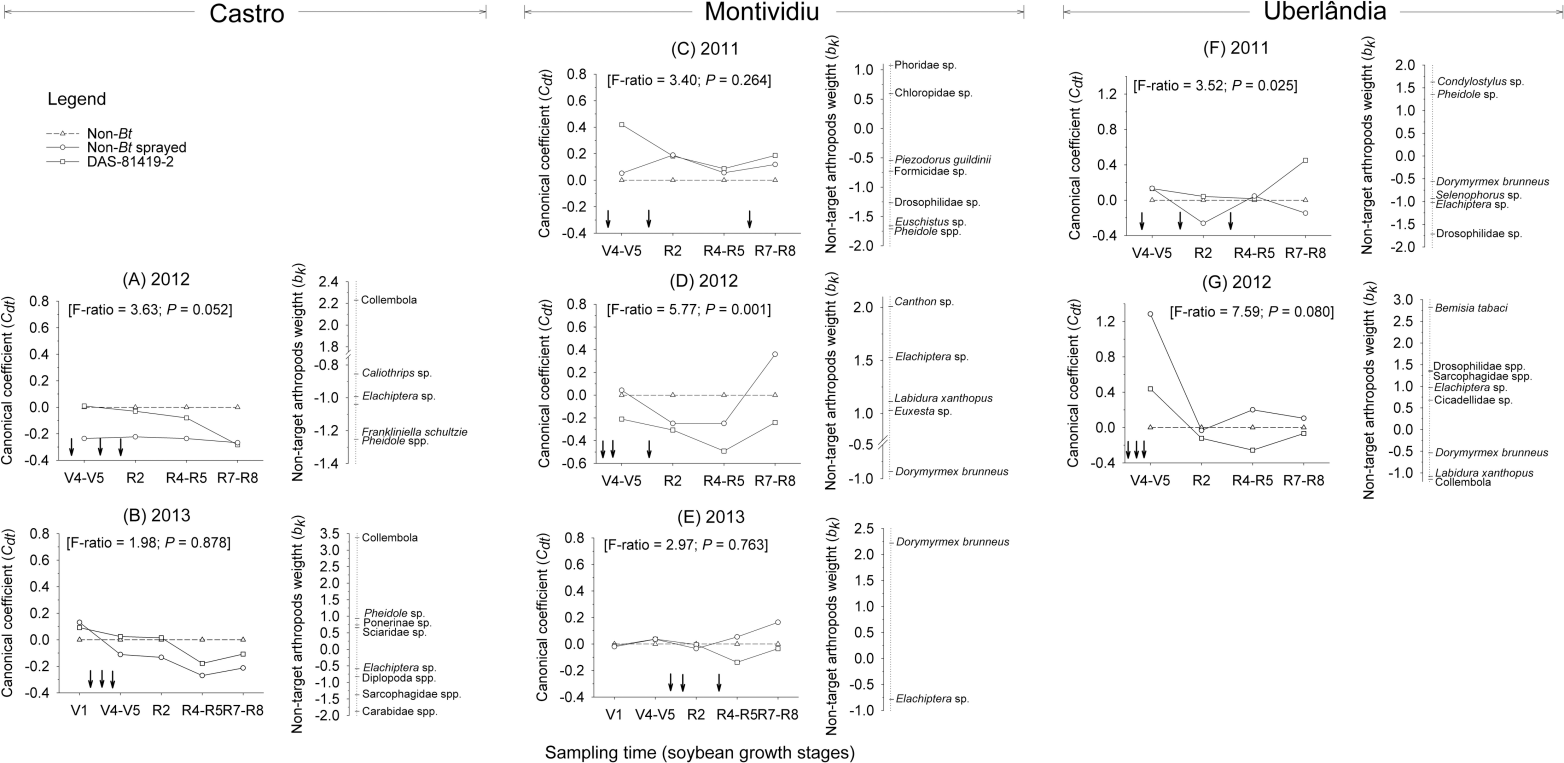
Principal Response Curves (PRC) indicating the effect of non-*Bt* soybean with insecticides sprayed and *Bt* soybean (DAS-81419-2) on the most representative non-target arthropods collected by Pitfall traps at three sites over two to three years in Brazil. Dotted line indicates the community response to the control treatment (non-*Bt* without insecticides). The non-target arthropods weight (*b*_*k*_) can be interpreted as the affinity of the taxon with the Principal Response Curves (*C*_*dt*_). Only taxa with *b*_*k*_ values greater than 0.5 and less than -0.5 are shown in the diagram. Following Monte Carlo permutation tests, *P*-values less than 0.05 indicate a statistically significant difference in community response between at least one of the treatments and the control. Down arrows (↓) indicate the time of insecticide application in the sprayed non-*Bt* treatment.

The redundancy analysis (RDA) of NTA pitfall trap data collected in Castro, followed by Monte Carlo tests, did not identify significant differences for either of the years in the study: 2012 (first axis: F = 3.63; P = 0.052) and 2013 (first axis: F = 1.98; P = 0.878) (S7 Table, Fig 3A and 3B). These results indicated that the soybean treatments did not affect non-target surface-dwelling groups within or across sampling time.

In Montividiu, the first axis was not significant in 2011 (F = 3.40; P = 0.264) (S7 Table, Fig 3C). Phorid and chloropid species (Diptera) tended to decrease in abundance over time, whereas *Euschistus* sp. (Hemiptera: Pentatomidae) and *Pheidole* spp. tended to increase (Fig 3C). In 2012, the first axis (F = 5.77; P = 0.001) was significant (S7 Table, Fig 3D). The first axis explained 47.9% of the variation of the surface-dwelling NTA community structure. Wherein 41.0% was explained by sampling time and 10.1% by soybean treatments (S7 Table). The PRC curves for sprayed non-*Bt* and DAS-81419-2 treatments were similar until the R4-R5 stages. In the R7-R8 stages, the DAS-81419-2 treatment presented a canonical coefficient higher than sprayed non-*Bt* treatment. Only the species *Dorymyrmex brunneus* (Forel, 1908) (Hymenoptera: Formicidae) did not follow the pattern of PRC curves (negative weight) of sprayed non-*Bt* and DAS-81419-2 treatments compared to the control (Fig 3D). In 2013, none of the axes were significant (first axis: F = 2.7; P = 0.763; all axes: F = 0.97; P = 0.547) (S7 Table, Fig 3E) indicating that the soybean treatments did not affect the surface-dwelling NTA community structure.

The analysis for the 2011 Uberlândia trial revealed significance only for first axis (first axis: F = 3.52; P = 0.025) (S7 Table, Fig 3F). During that year, the first axis explained 41.3% of the variation of the surface-dwelling NTA community structure, where 48.9% was explained by sampling time and 5.0% by soybean treatments (S7 Table). The PRC diagram revealed a late season increase in the abundance of *Condylostylus* sp. and *Pheidole* sp. in DAS-81419-2 plots and lower or variable abundances for *D*. *brunneus*, *Selenophorus* sp., *Elachiptera* sp. and Drosophilidae sp. in *Bt* and sprayed non-*Bt* treatments (Fig 3F). In 2012, the first axis was not significant (F = 7.59; P = 0.080) (S7 Table, Fig 3G). The analysis of organism weight indicated an expected increase in abundance of *D*. *brunneus*, *Labidura xanthopus* (Stal, 1855) (Dermaptera: Labiduridae) and Collembola in the treatments (Fig 3G).

**Fig 4:**
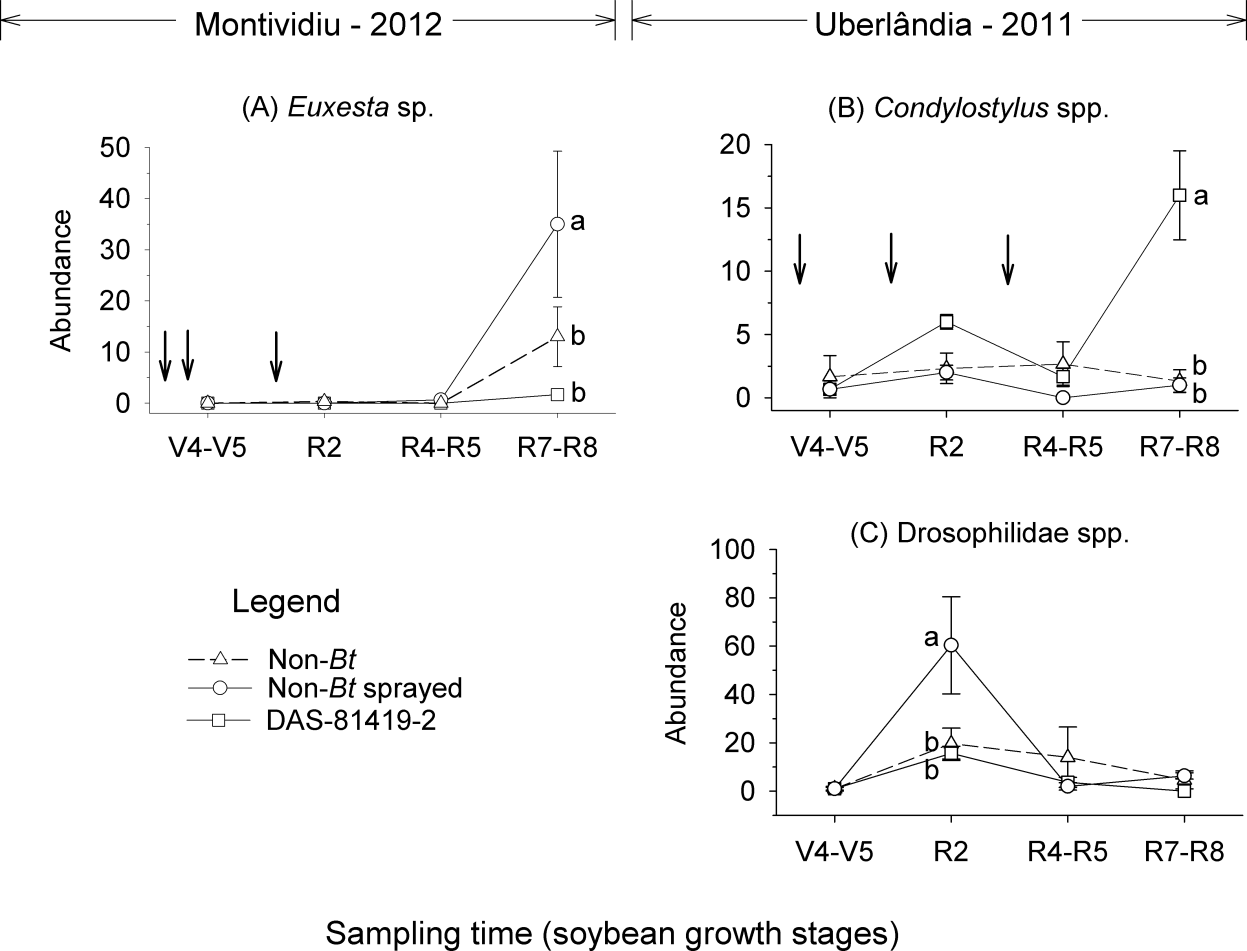
Population dynamics for non-target arthropod groups collected by Pitfall traps in non-*Bt* (with and without insecticides) and *Bt* (DAS-81419-2) soybean fields. Means (± SE) within sampling time followed by different letters are significantly different (Tukey’s test, α = 0.05). Taxa shown include those with taxon weights greater than 0.5 or less -0.5 which were associated with statistically significant Monte Carlo tests. Down arrows (↓) indicate the time of insecticide application in the sprayed non-*Bt* treatment.

The RM-ANOVA analysis was performed only for the sites and years in which the first axis was identified as significant. The results for Montividiu in 2012 demonstrated that the abundances of *Canthon* sp. (Coleoptera: Scarabaeidae) and *Elachiptera* sp. were higher in the non-sprayed non-*Bt* and sprayed non-*Bt* treatments compared with DAS-81419-2. Differences in abundance were observed for *Euxesta* sp. (Diptera: Otitidae) and *D*. *brunneus* among sampling times (Table 5). The interaction of sampling time and soybean treatment was significant only for *Euxesta* sp. where its abundance during the R7-R8 soybean stages was higher in the sprayed non-*Bt* treatment compared to the other treatments (Table 5, Fig 4A). The results for Uberlândia in 2011 identified higher abundance of *Condylostylus* spp. in the DAS-81419-2 treatment compared with the other treatments. Only *Pheidole* sp. was not influenced by sampling time (Table 5). Significant interactions of sampling time and soybean treatment were observed for *Condylostylus* spp. and Drosophilidae spp. (Table 5). The abundance of *Condylostylus* spp. during the R7-R8 soybean stages was higher in DAS-81419-2 plots compared with the other treatments (Fig 4B). The higher abundance of Drosophilidae spp. was observed at the R2 soybean stage in the sprayed non-*Bt* treatment (Fig 4C).

**Table 5:** Two-way repeated-measures ANOVA results for abundance of non-target arthropods with values weights greater than 0.5 and less -0.5 in the Principal Response Curves (PRC) analysis with first axis significant collected by Pitfall traps in non-*Bt* (with and without insecticides) and *Bt* (DAS-81419-2) soybean fields at two sites over two to three years in Brazil.

Site (year)	Non-target arthropods	Soybean treatments			Two-way ANOVA					
		Non-sprayed non-*Bt*	Sprayed non-*Bt*	DAS-81419-2	Soybean treat. (A)		Sampling time (B)		Interaction (A x B)	
					*F*	*P*	*F*	*P*	*F*	*P*
Montividiu (2012)	*Canthon* sp.	3.33 ± 0.99 a	5.75 ± 1.98 a	0.25 ± 0.18 b	17.01	**0.011**	1.22	0.381	2.67	0.070
	*Elachiptera* sp.	7.42 ± 1.60 a	7.42 ± 2.86 a	1.17 ± 0.36 b	46.87	**0.002**	1.97	0.220	1.13	0.400
	*Labidura xanthopus*	4.25 ± 1.19	3.42 ± 1.02	1.33 ± 0.39	3.99	0.112	2.10	0.202	1.82	0.177
	*Euxesta* sp.	3.33 ± 2.10	8.92 ± 3.95	0.42 ± 0.28	6.80	0.052	5.66	**0.035**	5.51	**0.006**
	*Dorymyrmex brunneus*	4.92 ± 1.92	6.42 ± 3.46	5.75 ± 2.29	0.24	0.795	8.97	**0.012**	2.22	0.113
Uberlândia (2011)	*Condylostylus* spp.	2.00 ± 0.63 b	0.92 ± 0.31 b	6.08 ± 1.99 a	19.44	**0.009**	5.57	**0.036**	9.56	**< 0.001**
	*Pheidole* sp.	6.75 ± 1.95	6.67 ± 1.50	17.00 ± 5.82	2.96	0.163	0.58	0.650	0.774	0.605
	*Dorymyrmex brunneus*	12.58 ± 3.91	6.42 ± 2.03	12.08 ± 3.60	0.99	0.445	5.04	**0.044**	1.45	0.276
	*Selenophorus* sp.	6.00 ± 1.54	26.17 ± 12.40	25.17 ± 11.97	1.13	0.409	25.89	**< 0.001**	2.57	0.077
	*Elachiptera* sp.	8.50 ± 2.55	8.00 ± 2.63	5.25 ± 2.61	0.51	0.635	8.48	**0.014**	0.48	0.809
	Drosophilidae spp.	9.83 ± 3.83	17.42 ± 8.65	5.08 ± 2.05	3.71	0.123	12.83	**0.005**	3.77	**0.024**

*P*-values highlighted in bold are statistically significant (α = 0.05).

Degrees of freedom: soybean fields = 2; sampling time = 3; interaction = 6; residual = 12.

Means (± SE) followed by different letters were significantly different within rows (Tukey’s test, α = 0.05).

## Discussion

The results obtained from field trials conducted over three years at multiple locations within key soybean producing regions in Brazil demonstrated that the NTA community was not adversely affected by *Bt* soybean event DAS-81419-2 and that the major differences among field treatments were mostly related to sampling date through the effect of soybean developmental stages on arthropod activity. In agricultural systems, the predominant factors that shape arthropod community composition include: regional climate and weather; cropping system, crop species and phenology (time effects); soil management practices such as tillage; crop management through the application of fertilizer or broad spectrum pesticides; and surrounding landscape diversity that influences NTAs directly and indirectly through prey/food-mediated effects [65]. Within the context of these factors, the effects of *Bt* proteins and other narrow-spectrum insect resistance traits on NTAs have been shown to be negligible [65–71]. Anthropogenic stress agents, like pesticides, are frequent components of agriculture ecosystems that potentially affect community structure and population dynamics through imparting lethal and/or sublethal effects on dominant species [72]. While lethality will deplete a species population directly via reducing its abundance, at least temporarily, sublethal effects are more difficult to detect in the field, and may lead to a range of subtle changes in the associated community, including impairment of species interactions, and eventual pest outbreaks, among others [72–75]. The same rationale is valid when assessing genetically modified crop plants that express insecticidal proteins, such as those including toxins derived from *Bacillus thuringiensis* (*Bt*) [72, 74]. Both lethal and sublethal effects on sensitive species are expected, primarily for target insect pest species, but also potentially for non-targeted species. Because the mode of action for *Bt* toxins is highly specific, requiring alkaline midgut pH and the presence of specific receptors on midgut epithelial cells, the range of potentially sensitive species has been shown to be very limited [76]. Indirect effects of *Bt* plants are expected due to a reduction in number or quality of target pests of the *Bt* proteins that serve as prey and/or hosts that attract predators and parasitoids [42]. Indirect effects may also arise if the *Bt* plants are more attractive or suitable for herbivores in the absence of significant damage from the target pests [18, 47, 48].

The number of studies evaluating the overall environmental impact of pesticides or *Bt* plants on arthropod assemblages (i.e., co-existing species of a given environment) and communities (i.e., interacting species of an assemblage) available under realistic field conditions is limited. Most available studies are short-term encompassing no more than a season or two and focus on a limited number of non-target species whose ecological benefits have been characterized, and thus are less likely to detect population or community-wide effects since these usually take longer to manifest and are strongly influenced by variation in environmental conditions beyond the presence of pesticides [74]. Here we reported a three-year study in three areas in different representative soybean-producing regions of Neotropical America designed to assess the potential impact of a *Bt* soybean variety, expressing the Cry1Ac and Cry1F proteins, as compared with its near (non-*Bt*) isoline with and without insecticide applications. The initial expectation was of a higher stress imposed by sprayed insecticides rather than by the *Bt* toxins based on previous studies on other crops [77–83]. Curiously, the effects of both *Bt* crops and sprayed insecticides were negligible in most of the instances investigated.

Results from diversity analyses were largely indistinguishable when the effects of the *Bt* proteins and insecticide applications were compared with non-sprayed non-*Bt* fields. This result was expected, as such generally low-resolution indices are less likely to provide evidence of environmental impact for insecticidal compounds [74]. Additionally, the time-dependent multivariate approach was used (i.e. Principal Response Curve method), and applied to higher taxa, focusing mainly at the genus and family levels. This multivariate approach indicated a statistically significant effect only in two out of seven instances where Moericke (aerial) traps collected arthropods associated with the soybean canopy, and in only two out of seven instances where pitfall traps collected epigeic arthropods (i.e., associated with the soil surface). In those instances, the *Bt* soybean treatment was associated with higher abundance of aerial non-target species both in Castro (2011) and Uberlândia (2011), and lower abundance in epigeic non-target arthropods in Montividiu (2012) and Uberlândia (2011), although in this last case an initial reduction in abundance was followed by an increase during the late reproductive period of the soybean crop, thus no consistent pattern was observed. Examination of successive sampling periods in each time-series demonstrated no sustained patterns. Furthermore, cross-referencing results with the complementing sampling method indicated no significant trends. Finally, abundance patterns were also not observed at all locations, indicating the few differences detected were not likely due to treatment effects.

Interpretation of results from multivariate analyses, such as the Principal Response Curve method, are benefitted when an optimal taxonomic resolution (species-, genus-, and/or family-level data) is achieved. The depth of taxonomic determinations may be guided by the potential for effects based on the known activity spectrum of the GM traits present in the crop, where family level data may suffice for most groups, and sub-family level determinations may aid in instances where taxonomic determinations for specific groups are practical and those groups are present in sufficient abundance to enable assessment.

The lack of insecticide effects in short-term studies of arthropod communities has also been detected in other ecosystems, particularly tropical agroecosystems [72, 84], where the cultivation system itself usually exhibits a more prominent effect and may buffer the potential insecticide impact [79–81, 85]. While *Bt* proteins are constitutively expressed in the crop throughout the growing season, there is a lack of significant short- and long-term impacts on arthropod assemblages, as reported for maize [15, 66], cotton [35, 47, 78, 86] and soybean [87]. Yu et al. [87] reported non-significant impact of *Bt*-soybean, but using general faunistic indexes rather than multivariate analyses with higher taxonomic resolution. Furthermore, Szénási et al. [88] also reported a lack of effect of *Bt* maize in central Europe using food-web analysis [74]. Thus, the relative lack of impact on the overall arthropod community or specific assemblages by *Bt* crops, as reported here, is in agreement with previous studies. Similar to previous studies, some statistically significant effects were detected in the *Bt* crop and separately in the non-*Bt* following insecticide application. The non-target arthropod herbivores cucurbit rootworm *D*. *speciosa*, the pollen beetle *A*. *variegatus*, the corn silk fly *Euxesta* sp., and leafhoppers were the main species for which differences were observed between *Bt* soybean and non-*Bt* soybean plots. Curcubit rootworm, a secondary pest species of soybean in Neotropical America [89], was reduced by insecticide application during the vegetative period up to the early reproductive stage. In contrast, the corn silk fly was more abundant in the insecticide treated plot in the late soybean reproductive period, while the pollen beetle and leafhoppers were more abundant in the *Bt* treatment during the mid and late soybean reproductive period, respectively. These species are highly mobile, and are not likely associated closely with treatment effects. None of these species are considered economic pests in the region [89], and their late increase is unlikely to affect the soybean arthropod community and more specifically the soybean-based food web. Rootworms (*D*. *speciosa*) are minor pests of soybean in Brazil and were not impacted by *Bt* soybean, but its suppression by insecticides may benefit soybean yield. Rather curious is the low incidence of stink bugs in the samples, a likely shortcoming from the sampling methods used, which efficiently capture a majority of aerial species but are not optimized for this non-target pest. This group is currently the major soybean pest in Brazil, Argentina, and Paraguay [89, 90] and it is not susceptible to current *Bt* soybean events, which only target lepidopteran pests. Thus, outbreaks of stink bugs are likely to occur even with the use of this technology and may be actually favored by its extensive adoption if stink-bug specific management tactics are not employed simultaneously.

Drosophilids and house flies were also collected in aerial traps. The number of drosophilids were significantly reduced in the *Bt* soybean plots at the early reproductive plant stage. The number of house flies were higher in *Bt* soybean early and late during the plant reproductive period. This outcome is unlikely to affect soybean yield and the associated arthropod community since these species are not relevant components in the soybean-based food web and given the effect was detected only in one field each, and in a single year. Examining natural enemies, where the *Bt* soybean was associated with higher incidence of parasitic tachinid flies, the long-legged fly *Condylostylus* sp., and the predatory ground beetle *L*. *concinna*. However, the incrementally higher abundance of these natural enemies were likely too late in the soybean reproductive period to impart any substantial benefit on soybean yield via biological control of economic pests [89].

In summary, a multi-year survey of the arthropod assemblage associated with soybean in three representative production regions indicated negligible short-term impact of *Bt* technology and sprayed insecticides. An effect was detected in only two instances and in isolated years restricted to few species of low relevance in the soybean-based food web which were unlikely to affect ecosystem processes or soybean yield. These findings are consistent with previous studies performed in cotton and soybean in the US [78, 87], maize in central Europe [88], China [91] and Brazil [92]. Use of *Bt* crops has reduced the need for insecticide applications in several cropping systems and survival of non-target arthropods has increased [18]. These non-target arthropods can include both beneficial species and other pest species. While an increase in predator, parasitoid, and pollinator populations’ benefits agriculture [93], an increased impact of pest hemipteran species has been documented in certain *Bt* cropping systems as a result of decreased broad-spectrum insecticide applications [94]. While such changes are predictable from the pre-commercial risk assessment of a *Bt* crop, their extent may take several cropping seasons to emerge and can be impacted by unrelated changes in the agricultural landscape that occur over time. In any case, insecticide applications targeted at emergent pests do not alter the environmental benefits of the *Bt* crops themselves. Although field-level research into non-target effects such as the present study can provide information about the ecology of GM fields, there is a considerable body of literature [2, 10, 16–18, 21, 95] that argues that such research is not necessary to perform regulatory risk assessment for GM crops. The present research supports that position in finding no unexpected adverse effects on NTA communities, a finding that was anticipated from lower tier NTA experiments conducted with higher concentrations of *Bt* proteins in controlled laboratory conditions [96, 97]. Assessments for GM crops that contain insect resistance traits could be expected to require field studies only when the results from lower tier hazard testing suggest the potential for adverse effects [16]. When applicable, post-commercialization assessments for *Bt* crops are expected to confirm the results of the research presented here, and offer additional opportunities to document the environmental safety of *Bt* crops and their contribution as a pest management tool [93]. Similarly, the results of ERAs that conclude no adverse effects of a novel trait(s) on non-target arthropods in one crop could be extended to other crops with the same or similar trait in a similar production system [19]. In the context of a tiered testing framework, field studies enable refinement of the risk assessment to address specific or unique considerations.

## Supporting information

S1 Table Gradient lengths via detrended correspondence analysis of the most representative non-target arthropods collected by Moericke (yellow pan) and pitfall traps in non-*Bt* (with and without insecticides) and *Bt* (DAS-81419-2) soybean fields at three sites over two to three years in Brazil. (DOC)

S2 Table Abundance and faunistic analysis results for the most representative non-target arthropods collected by Moericke traps (yellow pan) in non-*Bt* (with and without insecticides) and *Bt* (DAS-81419-2) soybean fields at three sites over two to three years in Brazil. (DOC)

S3 Table Abundance and faunistic analysis results for the most representative non-target arthropods collected by pitfall traps in non-*Bt* (with and without insecticides) and *Bt* (DAS-81419-2) soybean fields at three sites over two to three years in Brazil. (DOC)

S4 Table Diversity index values for non-target arthropods collected by Moericke traps (yellow pan) in non-*Bt* (with and without insecticides) and *Bt* (DAS-81419-2) soybean fields at three sites over two to three years in Brazil. (DOC)

S5 Table Summary of the redundancy analysis (RDA) of abundance of the most representative non-target arthropods collected by Moericke traps (yellow pan) in non-*Bt* (with and without insecticides) and *Bt* (DAS-81419-2) soybean fields at three sites over two to three years in Brazil. (DOC)

S6 Table Diversity index values for non-target arthropods collected by pitfall traps in non-*Bt* (with and without insecticides) and *Bt* (DAS-81419-2) soybean fields at three sites over two to three years in Brazil. (DOC)

S7 Table Summary of the redundancy analysis (RDA) of abundance of the most representative non-target arthropods collected by pitfall traps in non-*Bt* (with and without insecticides) and *Bt* (DAS-81419-2) soybean fields at three sites over two to three years in Brazil. (DOC)
