# Supplementary material for: Impact of transgenic soybean expressing Cry1Ac and Cry1F proteins on the non-target arthropod community associated with soybean in Brazil
Source: PLoS One. 2018 Feb 2;13(2):e0191567. doi: 10.1371/journal.pone.0191567 (PMC5796694; doi:10.1371/journal.pone.0191567)
Supplement: S1 Table — (DOC) [file pone.0191567.s001.doc]

**S1 Table. Gradient lengths via Detrended Correspondence Analysis of the most representative non-target arthropods collected by Moericke (yellow pan) and Pitfall traps in non-*Bt* (with and without insecticides) and *Bt* (DAS-81419-2) soybean fields at three sites over two to three years in Brazil.**

| Site | Year | Sampling method | |
| --- | --- | --- | --- |
|  |  | Moericke traps | Pitfall traps |
| Castro | 2012 | 0.89^a^ | 1.46 |
|  | 2013 | 1.66 | 2.13 |
| Montividiu | 2011 | 1.86 | 2.76 |
|  | 2012 | 1.38 | 1.78 |
|  | 2013 | 1.42 | 2.93 |
| Uberlândia | 2011 | 0.82 | 1.33 |
|  | 2012 | 1.45 | 1.62 |

^a^Gradient lengths shorter than 3.0 indicate appropriate application of linear response models in redundancy analysis (Lepš and Šmilauer (2003)).
