# Supplementary material for: Impact of transgenic soybean expressing Cry1Ac and Cry1F proteins on the non-target arthropod community associated with soybean in Brazil
Source: PLoS One. 2018 Feb 2;13(2):e0191567. doi: 10.1371/journal.pone.0191567 (PMC5796694; doi:10.1371/journal.pone.0191567)
Supplement: S2 Table — (DOC) [file pone.0191567.s002.doc]

**S2 Table. Abundance and faunistic analysis results for the most representative non-target arthropods collected by Moericke traps (yellow pan) in non-*Bt* (with and without insecticides) and *Bt* (DAS-81419-2) soybean fields at three sites over two to three years in Brazil.**

| Site (year) | Rank | Taxon | Functional group^1^ | No. individuals | | | Total | Faunistic indices^3^ | | | |
| --- | --- | --- | --- | --- | --- | --- | --- | --- | --- | --- | --- |
|  |  |  |  | Non-sprayed Non-*Bt* *t* | Sprayed Non-*Bt* | DAS-81419-2 |  | D | A | F | C |
| Castro  (2012) | 1 | *Astylus variegatus* | PHY | 3480 | 3247 | 6392 | 13119 | SD | SA | SF | W |
|  | 2 | *Frankliniella schultzei* | PHY | 3834 | 3013 | 3373 | 10220 | SD | SA | SF | W |
|  | 3 | *Frankliniella occidentalis* | PHY | 2756 | 2156 | 2006 | 6918 | SD | SA | SF | W |
|  | 4 | *Condylostylus* sp. | PRE | 1547 | 1166 | 2896 | 5609 | SD | SA | SF | W |
|  | 5 | *Elachiptera* spp. | PHY | 630 | 567 | 456 | 1653 | D | VA | VF | W |
|  | 6 | Drosophilidae spp. | DET | 373 | 378 | 289 | 1040 | D | VA | VF | W |
|  | 7 | Chrysomelidae spp. | PHY | 284 | 179 | 341 | 804 | D | VA | VF | W |
|  | 8 | Sarcophagidae spp. | PAR | 232 | 191 | 186 | 609 | D | VA | VF | W |
|  | 9 | *Megaselia* sp. | OMN | 142 | 129 | 159 | 430 | D | VA | VF | W |
|  | 10 | *Diabrotica* *speciosa* | PHY | 118 | 47 | 108 | 273 | D | VA | VF | W |
|  | 11 | Cicadellidae sp. | PHY | 32 | 64 | 50 | 146 | D | VA | VF | W |
|  | 12 | Tachinidae spp. | PAR | 26 | 38 | 71 | 135 | D | VA | VF | W |
|  | 13 | *Lebia concinna* | PRE | 36 | 26 | 64 | 126 | D | VA | VF | W |
|  | 14 | *Aphis* *gossypii* | PHY | 37 | 30 | 44 | 111 | D | VA | VF | W |
|  | 15 | *Musca domestica* | DET | 14 | 20 | 73 | 107 | D | VA | VF | W |
|  |  | Total |  | 13541 | 11251 | 16508 | 41300 |  |  |  |  |
|  |  | Others^4^ |  | 397 | 344 | 511 | 1252 |  |  |  |  |
|  |  | Total individuals |  | 13938 | 11595 | 17019 | 42552 |  |  |  |  |
|  |  | Total taxa |  | 86 | 83 | 107 | 133 |  |  |  |  |
| Castro  (2013) | 1 | *Condylostylus* spp. | PRE | 658 | 468 | 583 | 1709 | D | VA | VF | W |
|  | 2 | Sacophagidae spp. | PAR | 416 | 543 | 388 | 1347 | D | VA | VF | W |
|  | 3 | *Elachiptera* spp. | PHY | 519 | 362 | 351 | 1232 | D | VA | VF | W |
|  | 4 | *Caliothrips* spp. | PHY | 153 | 134 | 157 | 444 | D | VA | VF | W |
|  | 5 | *Frankliniella occidentalis* | PHY | 166 | 133 | 117 | 416 | D | VA | VF | W |
|  | 6 | Aphididae spp. | PHY | 89 | 146 | 179 | 414 | D | VA | VF | W |
|  | 7 | *Diabrotica speciosa* | PHY | 137 | 127 | 123 | 387 | D | VA | VF | W |
|  | 8 | Chloropidae spp. | PHY | 107 | 117 | 129 | 353 | D | VA | VF | W |
|  | 9 | *Pheidole* spp. | OMN | 62 | 49 | 231 | 342 | D | VA | VF | W |
|  | 10 | Sciaridae sp. | PHY | 94 | 134 | 89 | 317 | D | VA | VF | W |
|  | 11 | Drosophilidae spp. | DET | 110 | 121 | 77 | 308 | D | VA | VF | W |
|  | 12 | Collembola | DET | 58 | 45 | 64 | 167 | D | VA | VF | W |
|  | 13 | Empidoidea spp. | PRE | 66 | 41 | 49 | 156 | D | VA | VF | W |
|  | 14 | Pteromalidae spp. | PAR | 57 | 41 | 46 | 144 | D | VA | VF | W |
|  | 15 | *Astylus variegatus* | PHY | 59 | 25 | 41 | 125 | D | VA | VF | W |
|  | 16 | Miridae sp. | OMN | 23 | 44 | 23 | 90 | D | VA | VF | W |
|  | 17 | *Megaselia scalaris* | OMN | 48 | 22 | 19 | 89 | D | VA | VF | W |
|  | 18 | Cicadellidae spp. | PHY | 27 | 26 | 29 | 82 | D | VA | VF | W |
|  |  | Total |  | 2849 | 2578 | 2695 | 8122 |  |  |  |  |
|  |  | Others |  | 460 | 551 | 365 | 1376 |  |  |  |  |
|  |  | Total individuals |  | 3309 | 3129 | 3060 | 9498 |  |  |  |  |
|  |  | Total taxa |  | 135 | 148 | 112 | 207 |  |  |  |  |
| Montividiu  (2011) | 1 | *Condylostylus* spp. | PRE | 2308 | 2637 | 2271 | 7216 | SD | SA | SF | W |
|  | 2 | *Astylus variegatus* | PHY | 370 | 391 | 335 | 1096 | D | VA | VF | W |
|  | 3 | Sciaridae spp*.* | PHY | 208 | 179 | 155 | 542 | D | VA | VF | W |
|  | 4 | Drosophilidae spp*.* | DET | 113 | 157 | 144 | 414 | D | VA | VF | W |
|  | 5 | Aphididae spp*.* | PHY | 62 | 149 | 138 | 349 | D | VA | VF | W |
|  | 6 | *Elachiptera* sp*.* | PHY | 76 | 135 | 70 | 281 | D | VA | VF | W |
|  | 7 | *Coenosia* sp*.* | PRE | 93 | 102 | 82 | 277 | D | VA | VF | W |
|  | 8 | Cicadellidae spp. | PHY | 20 | 44 | 39 | 103 | D | VA | VF | W |
|  | 9 | Stratiomyidae sp. | PHY | 29 | 22 | 32 | 83 | D | VA | VF | W |
|  | 10 | Fulgoroidea sp. | PHY | 22 | 15 | 29 | 66 | D | VA | VF | W |
|  | 11 | Tachinidae spp. | PAR | 27 | 14 | 24 | 65 | D | VA | VF | W |
|  |  | Total |  | 3328 | 3845 | 3319 | 10492 |  |  |  |  |
|  |  | Others^4^ |  | 637 | 578 | 580 | 1795 |  |  |  |  |
|  |  | Total individuals |  | 3965 | 4423 | 3899 | 12287 |  |  |  |  |
|  |  | Total taxa |  | 97 | 109 | 99 | 150 |  |  |  |  |
| Montividiu  (2012) | 1 | *Bemisia tabaci* | PHY | 3403 | 3587 | 6643 | 13633 | SD | SA | SF | W |
|  | 2 | *Condylostylus* spp. | PRE | 1857 | 1736 | 2877 | 6470 | SD | SA | SF | W |
|  | 3 | *Elachiptera* spp. | PHY | 764 | 576 | 264 | 1604 | D | VA | VF | W |
|  | 4 | Drosophilidae spp. | DET | 298 | 216 | 122 | 636 | D | VA | VF | W |
|  | 5 | *Megaselia* sp. | OMN | 147 | 80 | 221 | 448 | D | VA | VF | W |
|  | 6 | Sarcophagidae spp. | PAR | 84 | 107 | 91 | 282 | D | VA | VF | W |
|  | 7 | Sciaridae spp. | PHY | 42 | 115 | 21 | 178 | D | VA | VF | W |
|  | 8 | Cicadellidae sp. | PHY | 54 | 35 | 64 | 153 | D | VA | VF | W |
|  | 9 | *Euxesta* spp. | PHY | 48 | 60 | 41 | 149 | D | VA | VF | W |
|  | 10 | Stratiomyidae sp. | PHY | 43 | 30 | 11 | 84 | D | VA | VF | W |
|  | 11 | Tachinidae spp. | PAR | 31 | 31 | 19 | 81 | D | VA | VF | W |
|  | 12 | *Dorymyrmex* *brunneus* | PRE | 9 | 31 | 40 | 80 | D | VA | VF | W |
|  |  | Total |  | 6780 | 6604 | 10414 | 23798 |  |  |  |  |
|  |  | Others |  | 378 | 377 | 340 | 1095 |  |  |  |  |
|  |  | Total individuals |  | 7158 | 6981 | 10754 | 24893 |  |  |  |  |
|  |  | Total taxa |  | 90 | 93 | 80 | 132 |  |  |  |  |
| Montividiu  (2013) | 1 | *Condylostylus* spp. | PRE | 2762 | 3072 | 3557 | 9391 | SD | SA | SF | W |
|  | 2 | Chloropidae spp. | PHY | 337 | 318 | 289 | 944 | D | VA | VF | W |
|  | 3 | Sciaridae sp. | PHY | 164 | 177 | 244 | 585 | D | VA | VF | W |
|  | 4 | Sarcophagidae spp. | PAR | 192 | 188 | 143 | 523 | D | VA | VF | W |
|  | 5 | Cicadellidae spp. | PHY | 136 | 148 | 149 | 433 | D | VA | VF | W |
|  | 6 | Tachinidae spp. | PAR | 92 | 108 | 101 | 301 | D | VA | VF | W |
|  | 7 | *Caliothrips* sp. | PHY | 97 | 72 | 67 | 236 | D | VA | VF | W |
|  | 8 | Drosophilidae spp. | DET | 72 | 52 | 69 | 193 | D | VA | VF | W |
|  | 9 | Halictidae sp. | OMN | 59 | 53 | 66 | 178 | D | VA | VF | W |
|  | 10 | Aphididae | PHY | 41 | 45 | 55 | 141 | D | VA | VF | W |
|  | 11 | Stratiomydae spp. | PHY | 38 | 42 | 49 | 129 | D | VA | VF | W |
|  | 12 | Chrysomelidae spp. | PHY | 46 | 34 | 36 | 116 | D | VA | VF | W |
|  | 13 | Psyllidae spp. (nymph) | PHY | 19 | 59 | 17 | 95 | D | VA | VF | W |
|  | 14 | *Coenosia* sp. | PRE | 24 | 29 | 22 | 75 | D | VA | VF | W |
|  | 15 | Mycetophilidae spp. | DET | 24 | 24 | 23 | 71 | D | VA | VF | W |
|  | 16 | Araneae spp. | PRE | 19 | 25 | 25 | 69 | D | VA | VF | W |
|  | 17 | Fulgoroidea spp. | PHY | 8 | 22 | 29 | 59 | D | VA | VF | W |
|  | 18 | Syrphidae spp. | PRE | 20 | 15 | 21 | 56 | D | VA | VF | W |
|  | 19 | *Euschistus heros* | PHY | 21 | 21 | 13 | 55 | D | VA | VF | W |
|  | 20 | Psyllidae spp. (adults) | PHY | 10 | 19 | 17 | 46 | D | VA | VF | W |
|  | 21 | Staphilinidae spp. | PRE | 12 | 8 | 25 | 45 | D | VA | VF | W |
|  |  | Total |  | 4193 | 4531 | 5017 | 13741 |  |  |  |  |
|  |  | Others |  | 1746 | 1617 | 1896 | 5259 |  |  |  |  |
|  |  | Total individuals |  | 5939 | 6148 | 6913 | 19000 |  |  |  |  |
|  |  | Total taxa |  | 141 | 132 | 147 | 218 |  |  |  |  |
| Uberlândia  (2011) | 1 | *Elachiptera* sp. | PHY | 1730 | 1841 | 871 | 4442 | D | VA | VF | W |
|  | 2 | *Condylostylus* sp. | PRE | 440 | 398 | 1108 | 1946 | D | VA | VF | W |
|  | 3 | *Selenophorus* sp. | OMN | 222 | 595 | 322 | 1139 | D | VA | VF | W |
|  | 4 | Drosophilidae sp. | DET | 427 | 437 | 212 | 1076 | D | VA | VF | W |
|  | 5 | Cicadellidae spp. | PHY | 214 | 168 | 541 | 923 | D | VA | VF | W |
|  | 6 | *Coenosia* sp. | PRE | 175 | 160 | 115 | 450 | D | VA | VF | W |
|  | 7 | Tachinidae spp. | PAR | 49 | 45 | 54 | 148 | D | VA | VF | W |
|  |  | Total |  | 3257 | 3644 | 3223 | 10124 |  |  |  |  |
|  |  | Others^4^ |  | 979 | 1041 | 1289 | 3309 |  |  |  |  |
|  |  | Total individuals |  | 4236 | 4685 | 4512 | 13433 |  |  |  |  |
|  |  | Total taxa |  | 149 | 149 | 137 | 222 |  |  |  |  |
| Uberlândia  (2012) | 1 | *Bemisia tabaci* | PHY | 34312 | 28957 | 26873 | 90142 | SD | SA | SF | W |
|  | 2 | *Condylostylus* sp. | PRE | 3300 | 4578 | 5061 | 12939 | SD | SA | SF | W |
|  | 3 | *Elachiptera* sp. | PHY | 4951 | 7344 | 540 | 12835 | SD | SA | SF | W |
|  | 4 | Chloropidae sp. | PHY | 588 | 2754 | 205 | 3547 | D | VA | VF | W |
|  | 5 | *Frankliniella schultzei* | PHY | 249 | 704 | 564 | 1517 | D | VA | VF | W |
|  | 6 | Sarcophagidae spp. | PAR | 457 | 157 | 379 | 993 | D | VA | VF | W |
|  | 7 | Cicadellidae spp. | PHY | 224 | 147 | 539 | 910 | D | VA | VF | W |
|  | 8 | Tachinidae spp. | PAR | 312 | 140 | 262 | 714 | D | VA | VF | W |
|  | 9 | Drosophilidae spp. | DET | 277 | 261 | 73 | 611 | D | VA | VF | W |
|  | 10 | *Coenosia* sp. | PRE | 64 | 49 | 310 | 423 | D | VA | VF | W |
|  | 11 | *Dorymyrmex brunneus* | PRE | 193 | 63 | 153 | 409 | D | VA | VF | W |
|  | 12 | *Eumecosomyia* sp. | PHY | 78 | 120 | 188 | 386 | D | VA | VF | W |
|  | 13 | *Euxesta* spp. | PHY | 80 | 116 | 111 | 307 | D | VA | VF | W |
|  | 14 | *Megaselia* sp. | OMN | 43 | 74 | 166 | 283 | D | VA | VF | W |
|  | 15 | Periscelididae sp. | PHY | 93 | 9 | 165 | 267 | D | VA | VF | W |
|  | 16 | *Allograpta* sp. | PHY | 77 | 13 | 158 | 248 | D | VA | VF | W |
|  | 17 | *Frankliniella occidentalis* | PHY | 35 | 76 | 98 | 209 | D | VA | VF | W |
|  | 18 | *Aphis gossypii* | PHY | 51 | 37 | 117 | 205 | D | VA | VF | W |
|  | 19 | Halictidae sp. | OMN | 70 | 43 | 87 | 200 | D | VA | VF | W |
|  | 20 | *Coenosia* sp. | PRE | 71 | 60 | 57 | 188 | D | VA | VF | W |
|  | 21 | Sciaridae spp. | PHY | 127 | 27 | 7 | 161 | D | VA | VF | W |
|  | 22 | *Diabrotica speciosa* | PHY | 53 | 42 | 49 | 144 | D | VA | VF | W |
|  | 23 | *Carpophilus* sp. | PHY | 41 | 59 | 34 | 134 | D | VA | VF | W |
|  | 24 | Fulgoroidea sp. | PHY | 40 | 20 | 60 | 120 | D | VA | VF | W |
|  | 25 | *Selenophorus* sp. | OMN | 79 | 26 | 12 | 117 | D | VA | VF | W |
|  | 26 | *Musca domestica* | DET | 21 | 21 | 71 | 113 | D | VA | VF | W |
|  | 27 | Pompilidae sp. | PRE | 54 | 26 | 27 | 107 | D | VA | VF | W |
|  |  | Total |  | 45940 | 45923 | 36366 | 128229 |  |  |  |  |
|  |  | Others |  | 1081 | 1490 | 1591 | 4162 |  |  |  |  |
|  |  | Total individuals |  | 47021 | 47413 | 37957 | 132391 |  |  |  |  |
|  |  | Total taxa |  | 193 | 175 | 209 | 271 |  |  |  |  |

^1^Detritivorous (DET), parasitoid (PAR), phytophagous (PHY), predator (PRE) or omnivorous (OMN).

^2^Total number of samples = 36.

^3^Faunistic indices: D = dominance (SD = super-dominant and D = dominant); A = abundance (SA = super-abundant and VA = very abundant); F = frequency (SF = super-frequent and VF = very frequent) and C = constancy (W = constant) according to the ANAFAU software (Moraes et al., 2003).

^4^Non-target arthropods that were not classified by the faunistic analysis as belonging to one of the classes cited above were added to ‘others’ and were not included in the Principal Response Curve (PRC) analysis.
