# Supplementary material for: Impact of transgenic soybean expressing Cry1Ac and Cry1F proteins on the non-target arthropod community associated with soybean in Brazil
Source: PLoS One. 2018 Feb 2;13(2):e0191567. doi: 10.1371/journal.pone.0191567 (PMC5796694; doi:10.1371/journal.pone.0191567)
Supplement: S3 Table — (DOC) [file pone.0191567.s003.doc]

**S3 Table. Abundance and faunistic analysis results for the most representative non-target arthropods collected by Pitfall traps in non-*Bt* (with and without insecticides) and *Bt* (DAS-81419-2) soybean fields at three sites over two to three years in Brazil.**

| Site (year) | Rank | Taxon | Functional group^1^ | No. individuals | | | | Total | Faunistic indices^3^ | | | |
| --- | --- | --- | --- | --- | --- | --- | --- | --- | --- | --- | --- | --- |
|  |  |  |  | Non-sprayed Non-*Bt* | Sprayed Non-*Bt* | DAS-81419-2 | |  | D | A | F | C |
| Castro  (2012) | 1 | Collembola | DET | 393 | 166 | | 199 | 758 | D | VA | VF | W |
|  | 2 | *Pheidole* spp. | OMN | 100 | 171 | 147 | | 418 | D | VA | VF | W |
|  | 3 | *Caliothrips* sp*.* | PHY | 142 | 118 | 106 | | 366 | D | VA | VF | W |
|  | 4 | Mollusca | PHY | 88 | 40 | 18 | | 146 | D | VA | VF | W |
|  | 5 | Araneae | PRE | 36 | 45 | 34 | | 115 | D | VA | VF | W |
|  | 6 | *Elachiptera* sp. | PHY | 18 | 38 | 29 | | 85 | D | VA | VF | W |
|  | 7 | Sciaridae sp. | PHY | 18 | 29 | 29 | | 76 | D | VA | VF | W |
|  | 8 | Annelida | DET | 17 | 29 | 29 | | 75 | D | VA | VF | W |
|  | 9 | *Frankliniella schultzie* | PHY | 12 | 36 | 11 | | 59 | D | VA | VF | W |
|  | 10 | Drosophilidae sp. | DET | 3 | 8 | 10 | | 21 | D | VA | VF | W |
|  |  | Total |  | 827 | 680 | 612 | | 2119 |  |  |  |  |
|  |  | Others^4^ |  | 70 | 98 | 122 | | 290 |  |  |  |  |
|  |  | Total individuals |  | 897 | 778 | 734 | | 2409 |  |  |  |  |
|  |  | Total taxa |  | 48 | 48 | 53 | | 75 |  |  |  |  |
| Castro  (2013) | 1 | Collembola sp. | DET | 314 | 212 | | 277 | 803 | SD | SA | SF | W |
|  | 2 | *Pheidole* spp. | OMN | 206 | 107 | 167 | | 480 | SD | SA | SF | W |
|  | 3 | Araneae spp. | PRE | 108 | 140 | 68 | | 316 | SD | SA | SF | W |
|  | 4 | Agromyzidae sp. | PHY | 55 | 31 | 45 | | 131 | D | VA | VF | W |
|  | 5 | Carabidae spp. | PRE | 29 | 27 | 15 | | 71 | D | VA | VF | W |
|  | 6 | Sarcophagidae spp. | PAR | 18 | 25 | 22 | | 65 | D | VA | VF | W |
|  | 7 | Sciaridae sp. | PHY | 14 | 16 | 11 | | 41 | D | VA | VF | W |
|  | 8 | *Elachiptera* sp. | PHY | 13 | 12 | 15 | | 40 | D | VA | VF | W |
|  | 9 | Ponerinae sp. | PRE | 7 | 6 | 24 | | 37 | D | VA | VF | W |
|  | 10 | Phoridae sp. | OMN | 19 | 11 | 5 | | 35 | D | VA | VF | W |
|  | 11 | Staphylinidae spp. | DET | 16 | 8 | 11 | | 35 | D | VA | VF | W |
|  | 12 | *Frankliniella* *schultzei* | PHY | 9 | 18 | 6 | | 33 | D | VA | VF | W |
|  | 13 | Drosophilidae spp. | DET | 8 | 7 | 8 | | 23 | D | VA | VF | W |
|  | 14 | *Diabrotica* *speciosa* | PHY | 9 | 6 | 7 | | 22 | D | VA | VF | W |
|  | 15 | Aphididae | PHY | 9 | 4 | 6 | | 19 | D | VA | VF | W |
|  | 16 | Diplopoda spp. | PHY | 6 | 7 | 4 | | 17 | D | VA | VF | W |
|  | 17 | Elateridae sp. | PRE | 7 | 6 | 4 | | 17 | D | VA | VF | W |
|  | 18 | *Condylostylus* sp. | PRE | 6 | 6 | 3 | | 15 | D | VA | VF | W |
|  | 19 | Cicadellidae spp. | PHY | 3 | 5 | 4 | | 12 | D | VA | VF | W |
|  | 20 | Pteromalidae spp. | PAR | 5 | 5 | 2 | | 12 | D | VA | VF | W |
|  |  | Total |  | 861 | 659 | 704 | | 2224 |  |  |  |  |
|  |  | Others |  | 91 | 80 | 73 | | 244 |  |  |  |  |
|  |  | Total individuals |  | 952 | 739 | 777 | | 2468 |  |  |  |  |
|  |  | Total taxa |  | 73 | 72 | | 73 | 127 |  |  |  |  |
| Montividiu  (2011) | 1 | *Pheidole* spp. | OMN | 109 | 59 | | 37 | 205 | D | VA | VF | W |
|  | 2 | Sciaridae spp. | PHY | 22 | 43 | 44 | | 109 | D | VA | VF | W |
|  | 3 | *Euschistus* sp. | PHY | 31 | 22 | 7 | | 60 | D | VA | VF | W |
|  | 4 | Drosophilidae sp. | DET | 40 | 17 | 1 | | 58 | D | VA | VF | W |
|  | 5 | Chloropidae sp. | PHY | 20 | 13 | 22 | | 55 | D | VA | VF | W |
|  | 6 | Phoridae sp. | OMN | 13 | 21 | 17 | | 51 | D | VA | VF | W |
|  | 7 | Formicidae sp. | OMN | 33 | 12 | 6 | | 51 | D | VA | VF | W |
|  | 8 | *Canthon* sp. | PRE | 15 | 25 | 7 | | 47 | D | VA | VF | W |
|  | 9 | *Gryllus assimilis* | PHY | 2 | 4 | 25 | | 31 | D | VA | VF | W |
|  | 10 | *Piezodorus guildinii* | PHY | 13 | 7 | 6 | | 26 | D | VA | VF | W |
|  |  | Total |  | 298 | 223 | 172 | | 693 |  |  |  |  |
|  |  | Others^4^ |  | 99 | 88 | 83 | | 270 |  |  |  |  |
|  |  | Total individuals |  | 397 | 311 | 255 | | 963 |  |  |  |  |
|  |  | Total taxa |  | 46 | 47 | | 45 | 72 |  |  |  |  |
| Montividiu  (2012) | 1 | *Bemisia tabaci* | PHY | 369 | 149 | | 98 | 616 | D | VA | VF | W |
|  | 2 | Sciaridae sp. | PHY | 200 | 187 | 127 | | 514 | D | VA | VF | W |
|  | 3 | *Dorymyrmex* *brunneus* | PRE | 59 | 77 | 69 | | 205 | D | VA | VF | W |
|  | 4 | *Elachiptera* sp. | PHY | 89 | 89 | 14 | | 192 | D | VA | VF | W |
|  | 5 | *Euxesta* sp. | PHY | 40 | 107 | 5 | | 152 | D | VA | VF | W |
|  | 6 | *Condylostylus* spp. | PRE | 138 | 3 | 2 | | 143 | D | VA | VF | W |
|  | 7 | *Canthon* sp. | PRE | 40 | 69 | 3 | | 112 | D | VA | VF | W |
|  | 8 | *Labidura* *xanthopus* | PRE | 51 | 41 | 16 | | 108 | D | VA | VF | W |
|  | 9 | *Anurogryllus* *muticus* | PHY | 31 | 32 | 20 | | 83 | D | VA | VF | W |
|  | 10 | *Pheidole* spp. | OMN | 2 | 55 | 19 | | 76 | D | VA | VF | W |
|  |  | Total |  | 1019 | 809 | 373 | | 2201 |  |  |  |  |
|  |  | Others |  | 275 | 206 | 126 | | 607 |  |  |  |  |
|  |  | Total individuals |  | 1294 | 1015 | 499 | | 2808 |  |  |  |  |
|  |  | Total taxa |  | 77 | 63 | 56 | | 104 |  |  |  |  |
| Montividiu  (2013) | 1 | *Pheidole* spp. | OMN | 27 | 14 | | 19 | 460 | D | VA | VF | W |
|  | 2 | Sciaridae spp. | PHY | 77 | 89 | 67 | | 233 | D | VA | VF | W |
|  | 3 | Araneae spp. | PRE | 25 | 23 | 24 | | 72 | D | VA | VF | W |
|  | 4 | *Dorymyrmex* *brunneus* | PRE | 14 | 32 | 0 | | 46 | D | VA | VF | W |
|  | 5 | *Elachiptera* sp. | PHY | 11 | 9 | 13 | | 33 | D | VA | VF | W |
|  | 6 | *Anurogryllus muticus* | PHY | 6 | 9 | 7 | | 22 | D | VA | VF | W |
|  |  | Total |  | 160 | 176 | 130 | | 466 |  |  |  |  |
|  |  | Others |  | 98 | 99 | 98 | | 295 |  |  |  |  |
|  |  | Total individuals |  | 258 | 275 | 228 | | 761 |  |  |  |  |
|  |  | Total taxa |  | 57 | 47 | | 48 | 88 |  |  |  |  |
| Uberlândia  (2011) | 1 | *Selenophorus* sp. | OMN | 72 | 314 | | 302 | 688 | D | VA | VF | W |
|  | 2 | Chloropidae sp. | PHY | 190 | 110 | 129 | | 429 | D | VA | VF | W |
|  | 3 | Drosophilidae spp. | DET | 118 | 209 | 61 | | 388 | D | VA | VF | W |
|  | 4 | *Dorymyrmex brunneus* | PRE | 151 | 77 | 145 | | 373 | D | VA | VF | W |
|  | 5 | *Pheidole* sp. | OMN | 81 | 80 | 204 | | 365 | D | VA | VF | W |
|  | 6 | *Elachiptera* sp. | PHY | 102 | 96 | 63 | | 261 | D | VA | VF | W |
|  | 7 | *Ladibura xantophus* | PRE | 70 | 90 | 66 | | 226 | D | VA | VF | W |
|  | 8 | *Galerita collaris* | PRE | 39 | 71 | 18 | | 128 | D | VA | VF | W |
|  | 9 | *Condylostylus* spp. | PRE | 24 | 11 | 73 | | 108 | D | VA | VF | W |
|  | 10 | Sciaridae spp. | PHY | 33 | 31 | 28 | | 92 | D | VA | VF | W |
|  |  | Total |  | 880 | 1089 | 1089 | | 3058 |  |  |  |  |
|  |  | Others^4^ |  | 167 | 259 | 273 | | 699 |  |  |  |  |
|  |  | Total individuals |  | 1047 | 1348 | 1362 | | 3757 |  |  |  |  |
|  |  | Total taxa |  | 66 | 68 | | 67 | 97 |  |  |  |  |
| Uberlândia  (2012) | 1 | Collembola | DET | 3302 | 1542 | | 2819 | 7663 | SD | SA | SF | W |
|  | 2 | *Bemisia tabaci* | PHY | 88 | 1218 | 1085 | | 2391 | SD | SA | SF | W |
|  | 3 | *Pheidole* spp. | OMN | 999 | 389 | 367 | | 1755 | SD | SA | SF | W |
|  | 4 | *Elachiptera* sp*.* | PHY | 347 | 342 | 97 | | 786 | D | VA | VF | W |
|  | 5 | *Labidura xanthopus* | PRE | 206 | 190 | 180 | | 576 | D | VA | VF | W |
|  | 6 | Sciaridae spp. | PHY | 168 | 164 | 169 | | 501 | D | VA | VF | W |
|  | 7 | *Dorymyrmex brunneus* | PRE | 113 | 101 | 275 | | 489 | D | VA | VF | W |
|  | 8 | Araneae | PRE | 43 | 260 | 39 | | 342 | D | VA | VF | W |
|  | 9 | Drosophilidae spp. | DET | 52 | 169 | 18 | | 239 | D | VA | VF | W |
|  | 10 | Sarcophagidae spp. | PAR | 7 | 122 | 70 | | 199 | D | VA | VF | W |
|  | 11 | *Euxesta* sp. | PHY | 34 | 100 | 43 | | 177 | D | VA | VF | W |
|  | 12 | *Anurogryllus muticus* | PHY | 34 | 44 | 34 | | 112 | D | VA | VF | W |
|  | 13 | Cicadellidae sp. | PHY | 6 | 54 | 38 | | 98 | D | VA | VF | W |
|  | 14 | Orthoptera (nymph) | PHY | 39 | 28 | 27 | | 94 | D | VA | VF | W |
|  | 15 | *Selenophorus* sp. | OMN | 25 | 42 | 24 | | 91 | D | VA | VF | W |
|  | 16 | *Dichroplus* sp. | PHY | 15 | 30 | 15 | | 60 | D | VA | VF | W |
|  |  | Total |  | 5478 | 4795 | 5300 | | 15573 |  |  |  |  |
|  |  | Others |  | 231 | 443 | 400 | | 1074 |  |  |  |  |
|  |  | Total individuals |  | 5709 | 5238 | 5700 | | 16647 |  |  |  |  |
|  |  | Total taxa |  | 88 | 91 | | 99 | 144 |  |  |  |  |

^1^Detritivorous (DET), parasitoid (PAR), phytophagous (PHY), predator (PRE) or omnivorous (OMN).

^2^Total number of samples = 36.

^3^Faunistic indices: D = dominance (SD = super-dominant and D = dominant); A = abundance (SA = super-abundant and VA = very abundant); F = frequency (SF = super-frequent and VF = very frequent) and C = constancy (W = constant) according to the ANAFAU software (Moraes et al., 2003).

^4^Non-target arthropods that were not classified by the faunistic analysis as belonging to one of the classes cited above were added to ‘others’ and were not included in the Principal Response Curve (PRC) analysis.
