# Supplementary material for: Impact of transgenic soybean expressing Cry1Ac and Cry1F proteins on the non-target arthropod community associated with soybean in Brazil
Source: PLoS One. 2018 Feb 2;13(2):e0191567. doi: 10.1371/journal.pone.0191567 (PMC5796694; doi:10.1371/journal.pone.0191567)
Supplement: S4 Table — (DOC) [file pone.0191567.s004.doc]

**S4 Table. Diversity index values for non-target arthropods collected by Moericke traps (yellow pan) in non-*Bt* (with and without insecticides) and *Bt* (DAS-81419-2) soybean fields at three sites over two to three years in Brazil.**

| Site (year) | Diversity indices^1^ | Soybean treatments | Sampling time (soybean growth stages) | | | | |
| --- | --- | --- | --- | --- | --- | --- | --- |
|  |  |  | V1 | V4 – V5 | R2 | R4 – R5 | R7 – R8 |
| Castro  (2012) | N | Non-sprayed Non-*Bt* | - | 1007.00 ± 33.20 a | 1783.00 ± 65.28 | 1139.67 ± 123.47 b | 716.33 ± 66.83 b |
|  |  | Sprayed Non-*Bt* | - | 581.67 ± 22.30 b | 1618.00 ± 18.77 | 1028.67 ± 54.82 b | 636.67 ± 70.17 b |
|  |  | DAS-81419-2 | - | 641.00 ± 7.23 b | 1996.00 ± 226.89 | 1765.00 ± 49.90 a | 1271.00 ± 85.94 a |
|  | S | Non-sprayed Non-*Bt* | - | 32.33 ± 4.67 | 28.67 ± 1.76 | 26.67 ± 0.88 | 24.67 ± 2.40 |
|  |  | Sprayed Non-*Bt* | - | 30.00 ± 1.53 | 24.67 ± 1.67 | 30.00 ± 0.58 | 22.67 ± 2.96 |
|  |  | DAS-81419-2 | - | 37.67 ± 1.33 | 28.33 ± 1.45 | 33.00 ± 1.15 | 30.67 ± 3.76 |
|  | H’ | Non-sprayed Non-*Bt* | - | 2.00 ± 0.10 b | 1.61 ± 0.01 | 1.84 ± 0.01 a | 1.98 ± 0.06 |
|  |  | Sprayed Non-*Bt* | - | 2.22 ± 0.03 a | 1.48 ± 0.02 | 1.91 ± 0.03 a | 1.93 ± 0.09 |
|  |  | DAS-81419-2 | - | 2.31 ± 0.05 a | 1.55 ± 0.08 | 1.53 ± 0.05 b | 1.94 ± 0.04 |
|  | D | Non-sprayed Non-*Bt* | - | 0.79 ± 0.02 | 0.71 ± 0.01 | 0.79 ± 0.01 a | 0.81 ± 0.01 |
|  |  | Sprayed Non-*Bt* | - | 0.84 ± 0.01 | 0.67 ± 0.00 | 0.79 ± 0.01 a | 0.80 ± 0.02 |
|  |  | DAS-81419-2 | - | 0.83 ± 0.02 | 0.68 ± 0.04 | 0.66 ± 0.03 b | 0.79 ± 0.01 |
|  | J | Non-sprayed Non-*Bt* | - | 0.24 ± 0.02 b | 0.17 ± 0.01 | 0.24 ± 0.01 a | 0.30 ± 0.02 a |
|  |  | Sprayed Non-*Bt* | - | 0.31 ± 0.02 a | 0.18 ± 0.01 | 0.23 ± 0.01 a | 0.31 ± 0.02 a |
|  |  | DAS-81419-2 | - | 0.27 ± 0.00 ab | 0.17 ± 0.02 | 0.14 ± 0.01 b | 0.23 ± 0.02 b |
| Castro  (2013) | N | Non-sprayed Non-*Bt* | 188.67 ± 68.79 | 157.33 ± 22.41 | 337.67 ± 26.26 | 219.67 ± 3.53 | 199.67 ± 89.24 |
|  |  | Sprayed Non-*Bt* | 224.33 ± 46.94 | 133.33 ± 26.67 | 264.33 ± 26.18 | 225.00 ± 11.02 | 196.00 ± 28.38 |
|  |  | DAS-81419-2 | 242.67 ± 47.61 | 123.00 ± 26.91 | 300.00 ± 13.01 | 182.33 ± 25.89 | 172.00 ± 75.29 |
|  | S | Non-sprayed Non-*Bt* | 29.33 ± 3.71 | 25.33 ± 2.19 | 37.67 ± 3.38 | 26.00 ± 2.08 | 28.67 ± 5.90 |
|  |  | Sprayed Non-*Bt* | 40.67 ± 1.76 | 25.00 ± 3.06 | 30.67 ± 0.67 | 25.33 ± 1.20 | 35.00 ± 4.00 |
|  |  | DAS-81419-2 | 26.00 ± 3.06 | 20.33 ± 3.53 | 30.33 ± 2.03 | 24.33 ± 2.03 | 28.67 ± 4.84 |
|  | H’ | Non-sprayed Non-*Bt* | 2.55 ± 0.19 | 2.40 ± 0.08 | 2.41 ± 0.05 | 2.26 ± 0.07 | 2.41 ± 0.16 |
|  |  | Sprayed Non-*Bt* | 2.95 ± 0.11 | 2.46 ± 0.12 | 2.45 ± 0.05 | 1.89 ± 0.07 | 2.73 ± 0.09 |
|  |  | DAS-81419-2 | 2.32 ± 0.20 | 2.22 ± 0.08 | 2.34 ± 0.14 | 2.20 ± 0.15 | 2.46 ± 0.11 |
|  | D | Non-sprayed Non-*Bt* | 0.86 ± 0.03 | 0.83 ± 0.01 | 0.83 ± 0.01 | 0.83 ± 0.01 | 0.83 ± 0.05 |
|  |  | Sprayed Non-*Bt* | 0.91 ± 0.01 | 0.85 ± 0.02 | 0.85 ± 0.02 | 0.71 ± 0.02 | 0.88 ± 0.01 |
|  |  | DAS-81419-2 | 0.83 ± 0.05 | 0.82 ± 0.00 | 0.83 ± 0.03 | 0.80 ± 0.03 | 0.85 ± 0.03 |
|  | J | Non-sprayed Non-*Bt* | 0.46 ± 0.07 | 0.44 ± 0.03 | 0.30 ± 0.02 | 0.37 ± 0.01 | 0.48 ± 0.21 |
|  |  | Sprayed Non-*Bt* | 0.47 ± 0.03 | 0.48 ± 0.06 | 0.38 ± 0.01 | 0.26 ± 0.02 | 0.44 ± 0.01 |
|  |  | DAS-81419-2 | 0.42 ± 0.09 | 0.47 ± 0.05 | 0.35 ± 0.03 | 0.38 ± 0.06 | 0.46 ± 0.15 |
| Montividiu  (2011) | N | Non-sprayed Non-*Bt* | - | 266.67 ± 46.26 | 147.33 ± 11.89 b | 207.67 ± 10.11 | 700.00 ± 12.29 |
|  |  | Sprayed Non-*Bt* | - | 296.00 ± 16.50 | 220.33 ± 51.60 ab | 216.67 ± 10.74 | 741.33 ± 24.17 |
|  |  | DAS-81419-2 | - | 225.33 ± 23.68 | 297.33 ± 12.81 a | 168.33 ± 14.71 | 608.67 ± 62.33 |
|  | S | Non-sprayed Non-*Bt* | - | 25.67 ± 2.73 | 27.33 ± 0.88 | 21.67 ± 2.19 | 22.00 ± 2.31 |
|  |  | Sprayed Non-*Bt* | - | 26.33 ± 0.88 | 23.33 ± 0.88 | 22.33 ± 1.86 | 24.67 ± 4.26 |
|  |  | DAS-81419-2 | - | 27.00 ± 1.00 | 29.33 ± 0.33 | 21.33 ± 1.76 | 23.33 ± 0.88 |
|  | H’ | Non-sprayed Non-*Bt* | - | 1.96 ± 0.26 | 2.33 ± 0.06 | 1.49 ± 0.03 | 1.19 ± 0.09 |
|  |  | Sprayed Non-*Bt* | - | 1.81 ± 0.09 | 1.84 ± 0.19 | 1.60 ± 0.04 | 1.23 ± 0.05 |
|  |  | DAS-81419-2 | - | 2.12 ± 0.06 | 1.75 ± 0.13 | 1.62 ± 0.10 | 1.35 ± 0.04 |
|  | D | Non-sprayed Non-*Bt* | - | 0.73 ± 0.09 | 0.82 ± 0.01 | 0.60 ± 0.02 | 0.51 ± 0.04 |
|  |  | Sprayed Non-*Bt* | - | 0.67 ± 0.04 | 0.72 ± 0.06 | 0.63 ± 0.02 | 0.53 ± 0.02 |
|  |  | DAS-81419-2 | - | 0.74 ± 0.02 | 0.62 ± 0.06 | 0.63 ± 0.04 | 0.57 ± 0.02 |
|  | J | Non-sprayed Non-*Bt* | - | 0.30 ± 0.07 | 0.38 ± 0.01 a | 0.21 ± 0.02 | 0.15 ± 0.01 |
|  |  | Sprayed Non-*Bt* | - | 0.24 ± 0.02 | 0.28 ± 0.04 b | 0.22 ± 0.01 | 0.14 ± 0.02 |
|  |  | DAS-81419-2 | - | 0.31 ± 0.01 | 0.20 ± 0.02 c | 0.24 ± 0.01 | 0.17 ± 0.01 |
| Montividiu  (2012) | N | Non-sprayed Non-*Bt* | - | 280.00 ± 94.17 | 174.00 ± 41.51 | 1481.33 ± 547.31 | 450.67 ± 63.56 |
|  |  | Sprayed Non-*Bt* | - | 185.67 ± 36.29 | 180.67 ± 13.17 | 1590.67 ± 312.86 | 370.00 ± 33.15 |
|  |  | DAS-81419-2 | - | 118.33 ± 20.51 | 83.33 ± 8.19 | 2940.67 ± 618.31 | 442.33 ± 28.17 |
|  | S | Non-sprayed Non-*Bt* | - | 24.67 ± 2.96 | 15.33 ± 0.88 | 26.00 ± 4.73 | 32.67 ± 0.88 |
|  |  | Sprayed Non-*Bt* | - | 19.00 ± 3.46 | 17.00 ± 3.00 | 35.00 ± 5.29 | 26.00 ± 2.00 |
|  |  | DAS-81419-2 | - | 18.33 ± 2.33 | 11.00 ± 0.58 | 25.33 ± 1.33 | 26.33 ± 2.73 |
|  | H’ | Non-sprayed Non-*Bt* | - | 2.05 ± 0.17 | 1.69 ± 0.11 | 1.15 ± 0.07 | 1.78 ± 0.12 |
|  |  | Sprayed Non-*Bt* | - | 2.08 ± 0.16 | 1.72 ± 0.07 | 1.07 ± 0.04 | 1.86 ± 0.15 |
|  |  | DAS-81419-2 | - | 2.12 ± 0.08 | 1.74 ± 0.05 | 0.83 ± 0.05 | 1.67 ± 0.02 |
|  | D | Non-sprayed Non-*Bt* | - | 0.79 ± 0.04 | 0.73 ± 0.06 | 0.52 ± 0.03 | 0.66 ± 0.07 |
|  |  | Sprayed Non-*Bt* | - | 0.82 ± 0.03 | 0.72 ± 0.00 | 0.47 ± 0.03 | 0.72 ± 0.03 |
|  |  | DAS-81419-2 | - | 0.81 ± 0.01 | 0.76 ± 0.02 | 0.42 ± 0.02 | 0.63 ± 0.02 |
|  | J | Non-sprayed Non-*Bt* | - | 0.34 ± 0.10 | 0.36 ± 0.06 | 0.14 ± 0.04 | 0.18 ± 0.02 |
|  |  | Sprayed Non-*Bt* | - | 0.44 ± 0.03 | 0.34 ± 0.03 | 0.09 ± 0.02 | 0.25 ± 0.02 |
|  |  | DAS-81419-2 | - | 0.47 ± 0.04 | 0.53 ± 0.05 | 0.09 ± 0.00 | 0.21 ± 0.02 |
| Montividiu  (2013) | N | Non-sprayed Non-*Bt* | 83.00 ± 5.00 | 199.00 ± 40.20 | 589.33 ± 60.08 | 393.33 ± 56.70 | 715.00 ± 53.95 |
|  |  | Sprayed Non-*Bt* | 121.33 ± 35.38 | 238.67 ± 55.95 | 483.67 ± 85.23 | 429.00 ± 25.74 | 776.67 ± 159.99 |
|  |  | DAS-81419-2 | 85.33 ± 8.41 | 361.33 ± 44.85 | 598.67 ± 78.12 | 470.67 ± 69.86 | 788.33 ± 107.98 |
|  | S | Non-sprayed Non-*Bt* | 20.00 ± 1.73 | 24.33 ± 2.03 b | 28.00 ± 2.89 a | 30.67 ± 0.33 | 41.00 ± 1.73 |
|  |  | Sprayed Non-*Bt* | 23.00 ± 1.00 | 28.67 ± 1.20 ab | 21.33 ± 2.73 b | 32.00 ± 3.46 | 41.33 ± 1.20 |
|  |  | DAS-81419-2 | 22.00 ± 1.15 | 33.33 ± 1.20 a | 29.33 ± 2.33 a | 32.33 ± 1.20 | 41.67 ± 3.18 |
|  | H’ | Non-sprayed Non-*Bt* | 2.12 ± 0.02 | 1.84 ± 0.06 | 1.36 ± 0.05 | 1.83 ± 0.06 | 1.44 ± 0.14 |
|  |  | Sprayed Non-*Bt* | 1.92 ± 0.34 | 1.97 ± 0.17 | 1.05 ± 0.10 | 1.77 ± 0.07 | 1.43 ± 0.19 |
|  |  | DAS-81419-2 | 2.23 ± 0.04 | 1.75 ± 0.10 | 1.21 ± 0.03 | 1.50 ± 0.23 | 1.40 ± 0.12 |
|  | D | Non-sprayed Non-*Bt* | 0.77 ± 0.01 | 0.69 ± 0.02 | 0.52 ± 0.02 | 0.66 ± 0.01 | 0.49 ± 0.05 |
|  |  | Sprayed Non-*Bt* | 0.67 ± 0.12 | 0.69 ± 0.06 | 0.40 ± 0.04 | 0.65 ± 0.02 | 0.50 ± 0.07 |
|  |  | DAS-81419-2 | 0.79 ± 0.02 | 0.60 ± 0.04 | 0.45 ± 0.01 | 0.53 ± 0.09 | 0.49 ± 0.05 |
|  | J | Non-sprayed Non-*Bt* | 0.42 ± 0.03 | 0.27 ± 0.04 | 0.14 ± 0.01 | 0.20 ± 0.01 | 0.10 ± 0.01 |
|  |  | Sprayed Non-*Bt* | 0.33 ± 0.10 | 0.26 ± 0.05 | 0.14 ± 0.03 | 0.19 ± 0.01 | 0.11 ± 0.02 |
|  |  | DAS-81419-2 | 0.43 ± 0.04 | 0.17 ± 0.02 | 0.12 ± 0.01 | 0.14 ± 0.03 | 0.10 ± 0.02 |
| Uberlândia  (2011) | N | Non-sprayed Non-*Bt* | - | 262.67 ± 79.64 ab | 612.67 ± 83.10 | 274.00 ± 60.21 | 262.67 ± 34.09 b |
|  |  | Sprayed Non-*Bt* | - | 344.67 ± 16.80 a | 731.00 ± 129.95 | 169.67 ± 5.24 | 316.33 ± 54.57 b |
|  |  | DAS-81419-2 | - | 161.00 ± 29.60 b | 562.33 ± 50.45 | 203.00 ± 16.50 | 577.67 ± 56.58 a |
|  | S | Non-sprayed Non-*Bt* | - | 36.67 ± 5.24 | 45.00 ± 5.03 | 39.00 ± 6.03 | 36.00 ± 3.06 c |
|  |  | Sprayed Non-*Bt* | - | 30.33 ± 2.03 | 45.33 ± 2.85 | 31.00 ± 0.58 | 50.67 ± 3.38 b |
|  |  | DAS-81419-2 | - | 26.33 ± 0.67 | 54.33 ± 4.91 | 35.00 ± 2.52 | 64.67 ± 2.73 a |
|  | H’ | Non-sprayed Non-*Bt* | - | 2.35 ± 0.08 a | 2.09 ± 0.04 b | 2.35 ± 0.08 | 2.58 ± 0.10 |
|  |  | Sprayed Non-*Bt* | - | 1.93 ± 0.14 b | 1.98 ± 0.09 b | 2.68 ± 0.01 | 2.73 ± 0.28 |
|  |  | DAS-81419-2 | - | 2.40 ± 0.08 a | 2.49 ± 0.10 a | 2.66 ± 0.09 | 2.50 ± 0.10 |
|  | D | Non-sprayed Non-*Bt* | - | 0.81 ± 0.02 ab | 0.72 ± 0.02 b | 0.81 ± 0.03 | 0.86 ± 0.01 |
|  |  | Sprayed Non-*Bt* | - | 0.73 ± 0.03 b | 0.72 ± 0.02 b | 0.88 ± 0.00 | 0.83 ± 0.05 |
|  |  | DAS-81419-2 | - | 0.85 ± 0.02 a | 0.82 ± 0.02 a | 0.88 ± 0.01 | 0.79 ± 0.03 |
|  | J | Non-sprayed Non-*Bt* | - | 0.30 ± 0.03 b | 0.18 ± 0.02 | 0.29 ± 0.06 b | 0.37 ± 0.03 a |
|  |  | Sprayed Non-*Bt* | - | 0.23 ± 0.02 b | 0.16 ± 0.01 | 0.47 ± 0.00 a | 0.32 ± 0.07 a |
|  |  | DAS-81419-2 | - | 0.42 ± 0.03 a | 0.22 ± 0.01 | 0.41 ± 0.01 a | 0.19 ± 0.01 b |
| Uberlândia  (2012) | N | Non-sprayed Non-*Bt* | - | 1289.00 ± 202.33 | 1902.67 ± 282.14 b | 6960.67 ± 1856.06 | 5521.67 ± 3005.98 ab |
|  |  | Sprayed Non-*Bt* | - | 468.00 ± 48.42 | 4484.33 ± 1391.63 a | 3610.33 ± 1008.11 | 7241.67 ± 1240.49 a |
|  |  | DAS-81419-2 | - | 1002.33 ± 338.68 | 1266.33 ± 146.03 b | 8081.33 ± 1710.17 | 2302.33 ± 403.19 b |
|  | S | Non-sprayed Non-*Bt* | - | 48.67 ± 4.81 | 65.33 ± 9.74 | 63.67 ± 5.04 | 66.00 ± 4.93 |
|  |  | Sprayed Non-*Bt* | - | 29.00 ± 4.51 | 52.00 ± 3.46 | 67.33 ± 1.33 | 69.33 ± 2.73 |
|  |  | DAS-81419-2 | - | 44.33 ± 6.44 | 64.33 ± 6.44 | 59.67 ± 1.76 | 79.00 ± 6.03 |
|  | H’ | Non-sprayed Non-*Bt* | - | 2.09 ± 0.21 | 1.43 ± 0.15 ab | 0.56 ± 0.13 | 1.24 ± 0.27 b |
|  |  | Sprayed Non-*Bt* | - | 1.96 ± 0.17 | 1.03 ± 0.15 b | 1.11 ± 0.21 | 1.20 ± 0.08 b |
|  |  | DAS-81419-2 | - | 2.14 ± 0.24 | 1.90 ± 0.22 a | 0.55 ± 0.03 | 1.90 ± 0.04 a |
|  | D | Non-sprayed Non-*Bt* | - | 0.76 ± 0.06 | 0.53 ± 0.06 | 0.18 ± 0.05 | 0.51 ± 0.11 |
|  |  | Sprayed Non-*Bt* | - | 0.75 ± 0.03 | 0.48 ± 0.07 | 0.43 ± 0.11 | 0.55 ± 0.02 |
|  |  | DAS-81419-2 | - | 0.76 ± 0.07 | 0.65 ± 0.05 | 0.20 ± 0.03 | 0.71 ± 0.01 |
|  | J | Non-sprayed Non-*Bt* | - | 0.17 ± 0.03 b | 0.07 ± 0.01 b | 0.03 ± 0.00 | 0.06 ± 0.02 |
|  |  | Sprayed Non-*Bt* | - | 0.25 ± 0.03 a | 0.06 ± 0.01 b | 0.05 ± 0.01 | 0.05 ± 0.01 |
|  |  | DAS-81419-2 | - | 0.20 ± 0.02 b | 0.11 ± 0.01 a | 0.03 ± 0.00 | 0.09 ± 0.01 |

^1^Diversity indices: abundance (N), richness (S), Shannon’s diversity index (H’), Simpson’s diversity index (D) and Pielou’s evenness index (J).

Means (± SE) of each site/year within sampling time followed by different letters are significantly different (Tukey’s test, α = 0.05).

Dashes indicate no data.
