# Supplementary material for: Impact of transgenic soybean expressing Cry1Ac and Cry1F proteins on the non-target arthropod community associated with soybean in Brazil
Source: PLoS One. 2018 Feb 2;13(2):e0191567. doi: 10.1371/journal.pone.0191567 (PMC5796694; doi:10.1371/journal.pone.0191567)
Supplement: S5 Table — (DOC) [file pone.0191567.s005.doc]

**S5 Table. Summary of the redundancy analysis (RDA) of abundance of the most representative non-target arthropods collected by Moericke traps (yellow pan) in non-*Bt* (with and without insecticides) and *Bt* (DAS-81419-2) soybean fields at three sites over two to three years in Brazil.**

| Site (year) |  | Axes | | | | Total variance |
| --- | --- | --- | --- | --- | --- | --- |
|  |  | First | Second | Third | Fourth |  |
| Castro  (2012) | Eigenvalues | 0.093 | 0.053 | 0.038 | 0.027 | 1.000 |
|  | Species-environment correlations | 0.968 | 0.842 | 0.827 | 0.806 |  |
|  | Cumulative percentage variance |  |  |  |  |  |
|  | of species data | 21.3 | 33.5 | 42.3 | 48.5 |  |
|  | of species-environment relation | 37.5 | 58.9 | 74.4 | 85.3 |  |
|  | Sum of all eigenvalues |  |  |  |  | 0.436 |
|  | Sum of all canonical eigenvalues |  |  |  |  | 0.248 |
|  | Partition of variability (%) |  |  |  |  |  |
|  | Sampling time | 56.3 |  |  |  |  |
|  | Soybean treatments | 8.8 |  |  |  |  |
|  | Permutation test for canonical axes^1^ | F-ratio | *P*-value |  |  |  |
|  | First axis | 6.49 | **0.001** |  |  |  |
|  | All axes | 3.95 | **0.001** |  |  |  |
| Castro  (2013) | Eigenvalues | 0.016 | 0.014 | 0.010 | 0.007 | 1.000 |
|  | Species-environment correlations | 0.673 | 0.536 | 0.533 | 0.477 |  |
|  | Cumulative percentage variance |  |  |  |  |  |
|  | of species data | 4.3 | 8.1 | 10.8 | 12.7 |  |
|  | of species-environment relation | 24.3 | 45.2 | 60.6 | 71.0 |  |
|  | Sum of all eigenvalues |  |  |  |  | 0.370 |
|  | Sum of all canonical eigenvalues |  |  |  |  | 0.066 |
|  | Partition of variability (%) |  |  |  |  |  |
|  | Sampling time | 63.0 |  |  |  |  |
|  | Soybean treatments | 1.3 |  |  |  |  |
|  | Permutation test for canonical axes | F-ratio | *P*-value |  |  |  |
|  | First axis | 1.36 | 0.994 |  |  |  |
|  | All axes | 0.65 | 0.981 |  |  |  |
| Montividiu  (2011) | Eigenvalues | 0.021 | 0.013 | 0.010 | 0.007 | 1.000 |
|  | Species-environment correlations | 0.756 | 0.668 | 0.623 | 0.582 |  |
|  | Cumulative percentage variance |  |  |  |  |  |
|  | of species data | 11.4 | 18.1 | 23.2 | 27.2 |  |
|  | of species-environment relation | 37.2 | 59.0 | 75.5 | 88.5 |  |
|  | Sum of all eigenvalues |  |  |  |  | 0.187 |
|  | Sum of all canonical eigenvalues |  |  |  |  | 0.058 |
|  | Partition of variability (%) |  |  |  |  |  |
|  | Sampling time | 81.3 |  |  |  |  |
|  | Soybean treatments | 1.5 |  |  |  |  |
|  | Permutation test for canonical axes^1^ | F-ratio | *P*-value |  |  |  |
|  | First axis | 3.09 | 0.082 |  |  |  |
|  | All axes | 1.33 | 0.057 |  |  |  |
| Montividiu  (2012) | Eigenvalues | 0.054 | 0.027 | 0.015 | 0.010 | 1.000 |
|  | Species-environment correlations | 0.698 | 0.750 | 0.615 | 0.706 |  |
|  | Cumulative percentage variance |  |  |  |  |  |
|  | of species data | 16.4 | 24.6 | 29.3 | 32.2 |  |
|  | of species-environment relation | 45.5 | 68.2 | 81.2 | 89.5 |  |
|  | Sum of all eigenvalues |  |  |  |  | 0.327 |
|  | Sum of all canonical eigenvalues |  |  |  |  | 0.118 |
|  | Partition of variability (%) |  |  |  |  |  |
|  | Sampling time | 67.3 |  |  |  |  |
|  | Soybean treatments | 5.8 |  |  |  |  |
|  | Permutation test for canonical axes | F-ratio | *P*-value |  |  |  |
|  | First axis | 4.71 | 0.091 |  |  |  |
|  | All axes | 1.69 | **0.020** |  |  |  |
| Montividiu  (2013) | Eigenvalues | 0.025 | 0.013 | 0.011 | 0.010 |  |
|  | Species-environment correlations | 0.766 | 0.822 | 0.754 | 0.675 |  |
|  | Cumulative percentage variance |  |  |  |  |  |
|  | of species data | 9.3 | 14.4 | 18.6 | 22.4 |  |
|  | of species-environment relation | 31.0 | 47.8 | 61.8 | 74.5 |  |
|  | Sum of all eigenvalues |  |  |  |  | 0.263 |
|  | Sum of all canonical eigenvalues |  |  |  |  | 0.079 |
|  | Partition of variability (%) |  |  |  |  |  |
|  | Sampling time | 73.7 |  |  |  |  |
|  | Soybean treatments | 1.0 |  |  |  |  |
|  | Permutation test for canonical axes | F-ratio | *P*-value |  |  |  |
|  | First axis | 3.09 | 0.161 |  |  |  |
|  | All axes | 1.29 | 0.056 |  |  |  |
| Uberlândia  (2011) | Eigenvalues | 0.120 | 0.045 | 0.014 | 0.008 | 1.000 |
|  | Species-environment correlations | 0.833 | 0.772 | 0.634 | 0.538 |  |
|  | Cumulative percentage variance |  |  |  |  |  |
|  | of species data | 29.4 | 40.5 | 43.9 | 45.9 |  |
|  | of species-environment relation | 62.3 | 85.9 | 93.1 | 97.4 |  |
|  | Sum of all eigenvalues |  |  |  |  | 0.407 |
|  | Sum of all canonical eigenvalues |  |  |  |  | 0.192 |
|  | Partition of variability (%) |  |  |  |  |  |
|  | Sampling time | 59.3 |  |  |  |  |
|  | Soybean treatments | 10.9 |  |  |  |  |
|  | Permutation test for canonical axes^1^ | F-ratio | *P*-value |  |  |  |
|  | First axis | 9.97 | **0.001** |  |  |  |
|  | All axes | 2.67 | **0.003** |  |  |  |
| Uberlândia  (2012) | Eigenvalues | 0.070 | 0.052 | 0.029 | 0.019 | 1.000 |
|  | Species-environment correlations | 0.860 | 0.780 | 0.740 | 0.827 |  |
|  | Cumulative percentage variance |  |  |  |  |  |
|  | of species data | 14.6 | 25.5 | 31.6 | 35.4 |  |
|  | of species-environment relation | 34.4 | 60.0 | 74.3 | 83.4 |  |
|  | Sum of all eigenvalues |  |  |  |  | 0.481 |
|  | Sum of all canonical eigenvalues |  |  |  |  | 0.204 |
|  | Partition of variability (%) |  |  |  |  |  |
|  | Sampling time | 51.9 |  |  |  |  |
|  | Soybean treatments | 8.4 |  |  |  |  |
|  | Permutation test for canonical axes | F-ratio | *P*-value |  |  |  |
|  | First axis | 4.11 | 0.084 |  |  |  |
|  | All axes | 2.22 | **0.005** |  |  |  |

^1^Permutation test by 999 Monte Carlo permutations (α = 0.05).

*P*-values highlighted in bold are statistically significant.

The abundance of non-target arthropods was log(x + 1) transformed before analysis.
