# Supplementary material for: Impact of transgenic soybean expressing Cry1Ac and Cry1F proteins on the non-target arthropod community associated with soybean in Brazil
Source: PLoS One. 2018 Feb 2;13(2):e0191567. doi: 10.1371/journal.pone.0191567 (PMC5796694; doi:10.1371/journal.pone.0191567)
Supplement: S6 Table — (DOC) [file pone.0191567.s006.doc]

**S6 Table. Diversity index values for non-target arthropods collected by Pitfall traps in non-*Bt* (with and without insecticides) and *Bt* (DAS-81419-2) soybean fields at three sites over two to three years in Brazil.**

| Site (year) | Diversity indices^1^ | Soybean treatments | Sampling time (soybean growth stages) | | | | |
| --- | --- | --- | --- | --- | --- | --- | --- |
|  |  |  | V1 | V4 – V5 | R2 | R4 – R5 | R7 – R8 |
| Castro  (2012) | N | Non-sprayed Non-*Bt* | - | 28.67 ± 7.51 | 31.00 ± 10.60 | 122.67 ± 20.95 | 116.67 ± 17.80 |
|  |  | Sprayed Non-*Bt* | - | 35.00 ± 4.04 | 30.00 ± 4.04 | 104.67 ± 6.64 | 89.67 ± 15.17 |
|  |  | DAS-81419-2 | - | 30.00 ± 8.19 | 38.33 ± 1.45 | 95.00 ± 16.64 | 81.33 ± 16.97 |
|  | S | Non-sprayed Non-*Bt* | - | 10.33 ± 1.20 | 8.67 ± 3.18 | 14.00 ± 2.31 | 12.33 ± 1.86 |
|  |  | Sprayed Non-*Bt* | - | 11.33 ± 0.88 | 9.00 ± 0.00 | 16.33 ± 1.45 | 14.00 ± 0.58 |
|  |  | DAS-81419-2 | - | 10.00 ± 1.15 | 11.00 ± 1.00 | 17.33 ± 2.60 | 17.67 ± 3.93 |
|  | H’ | Non-sprayed Non-*Bt* | - | 1.95 ± 0.04 | 1.65 ± 0.20 | 1.63 ± 0.16 | 1.31 ± 0.19 |
|  |  | Sprayed Non-*Bt* | - | 1.87 ± 0.02 | 1.61 ± 0.14 | 2.18 ± 0.03 | 1.96 ± 0.08 |
|  |  | DAS-81419-2 | - | 1.91 ± 0.12 | 1.76 ± 0.04 | 2.15 ± 0.15 | 2.18 ± 0.24 |
|  | D | Non-sprayed Non-*Bt* | - | 0.81 ± 0.01 | 0.74 ± 0.04 | 0.72 ± 0.03 b | 0.56 ± 0.05 b |
|  |  | Sprayed Non-*Bt* | - | 0.76 ± 0.01 | 0.68 ± 0.06 | 0.84 ± 0.00 a | 0.78 ± 0.02 a |
|  |  | DAS-81419-2 | - | 0.80 ± 0.04 | 0.73 ± 0.00 | 0.83 ± 0.02 a | 0.82 ± 0.04 a |
|  | J | Non-sprayed Non-*Bt* | - | 0.70 ± 0.07 | 0.69 ± 0.11 | 0.38 ± 0.04 | 0.31 ± 0.02 b |
|  |  | Sprayed Non-*Bt* | - | 0.58 ± 0.03 | 0.57 ± 0.08 | 0.55 ± 0.04 | 0.51 ± 0.05 a |
|  |  | DAS-81419-2 | - | 0.70 ± 0.12 | 0.53 ± 0.03 | 0.51 ± 0.03 | 0.53 ± 0.03 a |
| Castro  (2013) | N | Non-sprayed Non-*Bt* | 97.00 ± 13.32 | 137.33 ± 64.24 | 18.00 ± 6.24 | 33.33 ± 9.26 | 31.67 ± 8.11 |
|  |  | Sprayed Non-*Bt* | 117.33 ± 48.89 | 60.67 ± 12.55 | 14.00 ± 1.00 | 31.33 ± 6.01 | 23.00 ± 1.15 |
|  |  | DAS-81419-2 | 77.33 ± 17.98 | 99.00 ± 14.93 | 16.00 ± 5.13 | 39.33 ± 17.89 | 27.33 ± 17.37 |
|  | S | Non-sprayed Non-*Bt* | 18.00 ± 2.00 | 17.33 ± 3.76 | 10.33 ± 3.38 | 12.33 ± 3.33 | 11.67 ± 3.28 |
|  |  | Sprayed Non-*Bt* | 13.00 ± 4.16 | 16.33 ± 2.60 | 9.67 ± 1.20 | 11.00 ± 1.53 | 14.67 ± 0.67 |
|  |  | DAS-81419-2 | 15.33 ± 2.03 | 16.00 ± 1.73 | 8.33 ± 2.40 | 12.00 ± 1.73 | 9.00 ± 2.08 |
|  | H’ | Non-sprayed Non-*Bt* | 1.68 ± 0.12 | 1.88 ± 0.15 | 2.14 ± 0.29 | 2.04 ± 0.29 | 1.80 ± 0.47 |
|  |  | Sprayed Non-*Bt* | 1.26 ± 0.14 | 2.27 ± 0.05 | 2.18 ± 0.13 | 2.09 ± 0.19 | 2.47 ± 0.03 |
|  |  | DAS-81419-2 | 1.83 ± 0.20 | 1.72 ± 0.23 | 1.89 ± 0.29 | 1.88 ± 0.22 | 1.67 ± 0.10 |
|  | D | Non-sprayed Non-*Bt* | 0.65 ± 0.07 | 0.75 ± 0.04 | 0.86 ± 0.03 | 0.80 ± 0.07 | 0.69 ± 0.16 |
|  |  | Sprayed Non-*Bt* | 0.58 ± 0.05 | 0.85 ± 0.01 | 0.88 ± 0.02 | 0.84 ± 0.03 | 0.89 ± 0.01 |
|  |  | DAS-81419-2 | 0.73 ± 0.04 | 0.69 ± 0.09 | 0.81 ± 0.05 | 0.74 ± 0.11 | 0.73 ± 0.07 |
|  | J | Non-sprayed Non-*Bt* | 0.32 ± 0.07 | 0.42 ± 0.09 | 0.91 ± 0.02 | 0.69 ± 0.11 | 0.59 ± 0.13 |
|  |  | Sprayed Non-*Bt* | 0.34 ± 0.10 | 0.62 ± 0.09 | 0.93 ± 0.01 | 0.75 ± 0.04 | 0.81 ± 0.05 |
|  |  | DAS-81419-2 | 0.42 ± 0.05 | 0.37 ± 0.08 | 0.86 ± 0.04 | 0.62 ± 0.18 | 0.68 ± 0.18 |
| Montividiu  (2011) | N | Non-sprayed Non-*Bt* | - | 45.00 ± 8.50 | 23.00 ± 1.73 | 22.33 ± 0.67 | 42.00 ± 7.02 |
|  |  | Sprayed Non-*Bt* | - | 24.67 ± 3.76 | 12.67 ± 4.81 | 29.33 ± 6.84 | 37.00 ± 18.56 |
|  |  | DAS-81419-2 | - | 28.33 ± 8.09 | 10.33 ± 2.40 | 18.67 ± 7.88 | 27.67 ± 10.37 |
|  | S | Non-sprayed Non-*Bt* | - | 12.33 ± 0.88 | 7.67 ± 0.33 | 8.67 ± 0.88 | 11.00 ± 1.00 |
|  |  | Sprayed Non-*Bt* | - | 12.00 ± 0.58 | 7.33 ± 1.86 | 10.00 ± 1.53 | 11.33 ± 1.86 |
|  |  | DAS-81419-2 | - | 10.67 ± 2.60 | 6.67 ± 0.67 | 4.67 ± 1.67 | 9.33 ± 1.86 |
|  | H’ | Non-sprayed Non-*Bt* | - | 1.94 ± 0.24 | 1.76 ± 0.11 | 1.90 ± 0.11 | 1.71 ± 0.13 |
|  |  | Sprayed Non-*Bt* | - | 2.23 ± 0.10 | 1.85 ± 0.23 | 1.79 ± 0.31 | 2.00 ± 0.11 |
|  |  | DAS-81419-2 | - | 1.96 ± 0.24 | 1.79 ± 0.08 | 1.12 ± 0.34 | 1.91 ± 0.15 |
|  | D | Non-sprayed Non-*Bt* | - | 0.76 ± 0.08 | 0.79 ± 0.03 | 0.82 ± 0.02 | 0.72 ± 0.05 |
|  |  | Sprayed Non-*Bt* | - | 0.86 ± 0.02 | 0.82 ± 0.04 | 0.73 ± 0.11 | 0.80 ± 0.04 |
|  |  | DAS-81419-2 | - | 0.81 ± 0.05 | 0.81 ± 0.01 | 0.57 ± 0.13 | 0.81 ± 0.02 |
|  | J | Non-sprayed Non-*Bt* | - | 0.59 ± 0.11 | 0.76 ± 0.05 | 0.78 ± 0.02 | 0.51 ± 0.02 |
|  |  | Sprayed Non-*Bt* | - | 0.78 ± 0.06 | 0.92 ± 0.01 | 0.64 ± 0.11 | 0.69 ± 0.11 |
|  |  | DAS-81419-2 | - | 0.71 ± 0.06 | 0.90 ± 0.03 | 0.75 ± 0.10 | 0.75 ± 0.03 |
| Montividiu  (2012) | N | Non-sprayed Non-*Bt* | - | 48.33 ± 10.41 | 47.33 ± 4.81 | 245.67 ± 145.44 | 90.00 ± 2.08 |
|  |  | Sprayed Non-*Bt* | - | 58.67 ± 16.60 | 38.00 ± 16.17 | 121.33 ± 9.91 | 120.33 ± 46.25 |
|  |  | DAS-81419-2 | - | 37.00 ± 5.20 | 18.00 ± 4.51 | 59.67 ± 19.22 | 51.67 ± 9.74 |
|  | S | Non-sprayed Non-*Bt* | - | 13.67 ± 0.33 | 18.00 ± 0.58 | 16.00 ± 2.00 | 21.33 ± 3.38 |
|  |  | Sprayed Non-*Bt* | - | 16.00 ± 2.08 | 14.00 ± 2.00 | 15.33 ± 3.33 | 20.67 ± 2.60 |
|  |  | DAS-81419-2 | - | 14.00 ± 1.00 | 8.33 ± 1.76 | 9.67 ± 0.33 | 15.00 ± 1.53 |
|  | H’ | Non-sprayed Non-*Bt* | - | 1.91 ± 0.13 | 2.58 ± 0.08 | 1.80 ± 0.29 | 2.55 ± 0.16 |
|  |  | Sprayed Non-*Bt* | - | 2.09 ± 0.12 | 2.22 ± 0.06 | 1.79 ± 0.21 | 2.35 ± 0.06 |
|  |  | DAS-81419-2 | - | 2.08 ± 0.15 | 1.86 ± 0.23 | 1.58 ± 0.14 | 2.21 ± 0.23 |
|  | D | Non-sprayed Non-*Bt* | - | 0.74 ± 0.05 | 0.90 ± 0.01 | 0.73 ± 0.09 | 0.89 ± 0.02 |
|  |  | Sprayed Non-*Bt* | - | 0.80 ± 0.03 | 0.85 ± 0.02 | 0.74 ± 0.05 | 0.85 ± 0.02 |
|  |  | DAS-81419-2 | - | 0.79 ± 0.04 | 0.80 ± 0.05 | 0.73 ± 0.04 | 0.84 ± 0.04 |
|  | J | Non-sprayed Non-*Bt* | - | 0.51 ± 0.08 | 0.73 ± 0.04 | 0.44 ± 0.15 | 0.62 ± 0.03 |
|  |  | Sprayed Non-*Bt* | - | 0.53 ± 0.10 | 0.70 ± 0.12 | 0.41 ± 0.04 | 0.52 ± 0.07 |
|  |  | DAS-81419-2 | - | 0.58 ± 0.04 | 0.81 ± 0.02 | 0.52 ± 0.10 | 0.62 ± 0.09 |
| Montividiu  (2013) | N | Non-sprayed Non-*Bt* | 7.00 ± 2.31 | 13.33 ± 3.84 | 13.33 ± 4.37 | 17.00 ± 8.74 | 35.33 ± 1.86 |
|  |  | Sprayed Non-*Bt* | 12.00 ± 2.00 | 7.67 ± 1.20 | 14.67 ± 1.67 | 20.33 ± 4.70 | 37.00 ± 12.90 |
|  |  | DAS-81419-2 | 11.00 ± 1.15 | 8.00 ± 1.53 | 11.33 ± 1.45 | 16.67 ± 9.67 | 29.00 ± 12.53 |
|  | S | Non-sprayed Non-*Bt* | 4.67 ± 0.88 | 7.67 ± 0.33 | 9.00 ± 1.00 | 8.00 ± 2.89 | 10.00 ± 2.65 |
|  |  | Sprayed Non-*Bt* | 5.33 ± 1.20 | 5.00 ± 0.58 | 10.00 ± 0.00 | 8.33 ± 1.45 | 9.67 ± 1.67 |
|  |  | DAS-81419-2 | 6.67 ± 1.20 | 6.33 ± 0.88 | 7.67 ± 1.20 | 7.33 ± 2.85 | 8.33 ± 1.86 |
|  | H’ | Non-sprayed Non-*Bt* | 1.44 ± 0.19 | 1.83 ± 0.14 | 2.02 ± 0.03 | 1.70 ± 0.38 | 1.55 ± 0.45 |
|  |  | Sprayed Non-*Bt* | 1.42 ± 0.27 | 1.44 ± 0.11 | 2.16 ± 0.05 | 1.84 ± 0.24 | 1.67 ± 0.19 |
|  |  | DAS-81419-2 | 1.68 ± 0.15 | 1.76 ± 0.12 | 1.91 ± 0.19 | 1.66 ± 0.26 | 1.34 ± 0.08 |
|  | D | Non-sprayed Non-*Bt* | 0.74 ± 0.04 | 0.80 ± 0.06 | 0.84 ± 0.02 | 0.75 ± 0.09 | 0.62 ± 0.14 |
|  |  | Sprayed Non-*Bt* | 0.70 ± 0.08 | 0.72 ± 0.03 | 0.86 ± 0.01 | 0.79 ± 0.07 | 0.72 ± 0.06 |
|  |  | DAS-81419-2 | 0.77 ± 0.03 | 0.81 ± 0.02 | 0.83 ± 0.04 | 0.77 ± 0.04 | 0.59 ± 0.03 |
|  | J | Non-sprayed Non-*Bt* | 0.94 ± 0.04 | 0.84 ± 0.12 | 0.86 ± 0.09 | 0.82 ± 0.09 | 0.54 ± 0.13 |
|  |  | Sprayed Non-*Bt* | 0.83 ± 0.02 | 0.86 ± 0.01 | 0.87 ± 0.04 | 0.79 ± 0.08 | 0.60 ± 0.15 |
|  |  | DAS-81419-2 | 0.83 ± 0.03 | 0.94 ± 0.02 | 0.90 ± 0.04 | 0.84 ± 0.08 | 0.49 ± 0.08 |
| Uberlândia  (2011) | N | Non-sprayed Non-*Bt* | - | 86.00 ± 11.53 | 136.00 ± 15.31 | 70.00 ± 21.78 | 57.00 ± 6.81 |
|  |  | Sprayed Non-*Bt* | - | 87.33 ± 23.56 | 253.00 ± 55.43 | 45.00 ± 8.66 | 64.00 ± 10.82 |
|  |  | DAS-81419-2 | - | 89.67 ± 26.42 | 191.00 ± 47.09 | 87.00 ± 26.85 | 86.33 ± 15.90 |
|  | S | Non-sprayed Non-*Bt* | - | 21.00 ± 2.00 a | 18.00 ± 1.00 b | 12.00 ± 1.00 b | 18.00 ± 1.15 |
|  |  | Sprayed Non-*Bt* | - | 17.33 ± 3.33 ab | 25.00 ± 1.00 a | 15.67 ± 2.03 b | 17.00 ± 0.00 |
|  |  | DAS-81419-2 | - | 15.67 ± 3.18 b | 24.67 ± 1.45 a | 21.00 ± 3.79 a | 18.00 ± 1.00 |
|  | H’ | Non-sprayed Non-*Bt* | - | 2.42 ± 0.14 | 2.11 ± 0.13 | 1.85 ± 0.11 | 2.33 ± 0.11 |
|  |  | Sprayed Non-*Bt* | - | 2.20 ± 0.10 | 2.15 ± 0.12 | 2.30 ± 0.12 | 2.30 ± 0.10 |
|  |  | DAS-81419-2 | - | 1.92 ± 0.13 | 2.37 ± 0.20 | 2.37 ± 0.47 | 2.03 ± 0.11 |
|  | D | Non-sprayed Non-*Bt* | - | 0.86 ± 0.02 | 0.82 ± 0.04 | 0.77 ± 0.03 | 0.85 ± 0.02 |
|  |  | Sprayed Non-*Bt* | - | 0.84 ± 0.02 | 0.80 ± 0.03 | 0.86 ± 0.02 | 0.85 ± 0.02 |
|  |  | DAS-81419-2 | - | 0.77 ± 0.04 | 0.83 ± 0.04 | 0.81 ± 0.11 | 0.78 ± 0.03 |
|  | J | Non-sprayed Non-*Bt* | - | 0.55 ± 0.06 | 0.47 ± 0.07 | 0.54 ± 0.07 | 0.58 ± 0.04 |
|  |  | Sprayed Non-*Bt* | - | 0.54 ± 0.04 | 0.35 ± 0.05 | 0.65 ± 0.05 | 0.59 ± 0.06 |
|  |  | DAS-81419-2 | - | 0.48 ± 0.12 | 0.45 ± 0.09 | 0.57 ± 0.14 | 0.43 ± 0.07 |
| Uberlândia  (2012) | N | Non-sprayed Non-*Bt* | - | 848.33 ± 168.50 | 431.67 ± 56.53 | 353.33 ± 78.83 | 269.67 ± 42.94 |
|  |  | Sprayed Non-*Bt* | - | 747.00 ± 160.82 | 480.00 ± 73.58 | 321.33 ± 43.85 | 197.67 ± 16.60 |
|  |  | DAS-81419-2 | - | 1098.00 ± 266.07 | 362.67 ± 55.92 | 183.67 ± 49.10 | 255.67 ± 33.15 |
|  | S | Non-sprayed Non-*Bt* | - | 18.00 ± 1.00 | 21.67 ± 3.18 | 24.00 ± 1.00 | 22.67 ± 4.33 |
|  |  | Sprayed Non-*Bt* | - | 32.00 ± 3.79 | 19.33 ± 1.76 | 22.33 ± 1.86 | 21.00 ± 0.58 |
|  |  | DAS-81419-2 | - | 28.67 ± 6.49 | 24.33 ± 4.06 | 21.33 ± 2.03 | 20.00 ± 2.89 |
|  | H’ | Non-sprayed Non-*Bt* | - | 0.74 ± 0.13 | 1.45 ± 0.13 | 1.91 ± 0.27 | 1.68 ± 0.19 |
|  |  | Sprayed Non-*Bt* | - | 1.87 ± 0.18 | 1.34 ± 0.07 | 2.03 ± 0.22 | 2.20 ± 0.09 |
|  |  | DAS-81419-2 | - | 1.02 ± 0.27 | 1.49 ± 0.17 | 2.09 ± 0.10 | 1.80 ± 0.25 |
|  | D | Non-sprayed Non-*Bt* | - | 0.29 ± 0.05 | 0.61 ± 0.05 | 0.73 ± 0.10 | 0.64 ± 0.07 |
|  |  | Sprayed Non-*Bt* | - | 0.71 ± 0.07 | 0.55 ± 0.03 | 0.77 ± 0.08 | 0.81 ± 0.03 |
|  |  | DAS-81419-2 | - | 0.37 ± 0.10 | 0.56 ± 0.04 | 0.80 ± 0.03 | 0.72 ± 0.06 |
|  | J | Non-sprayed Non-*Bt* | - | 0.12 ± 0.02 | 0.21 ± 0.03 | 0.30 ± 0.06 | 0.26 ± 0.06 |
|  |  | Sprayed Non-*Bt* | - | 0.21 ± 0.04 | 0.20 ± 0.03 | 0.35 ± 0.05 | 0.43 ± 0.03 |
|  |  | DAS-81419-2 | - | 0.10 ± 0.01 | 0.19 ± 0.02 | 0.39 ± 0.05 | 0.32 ± 0.06 |

^1^Diversity indices: abundance (N), richness (S), Shannon’s diversity index (H’), Simpson’s diversity index (D) and Pielou’s evenness index (J).

Means (± SE) of each site/year within sampling time followed by different letters are significantly different (Tukey’s test, α = 0.05).

Dashes indicate no data.
