# Supplementary material for: Impact of transgenic soybean expressing Cry1Ac and Cry1F proteins on the non-target arthropod community associated with soybean in Brazil
Source: PLoS One. 2018 Feb 2;13(2):e0191567. doi: 10.1371/journal.pone.0191567 (PMC5796694; doi:10.1371/journal.pone.0191567)
Supplement: S7 Table — (DOC) [file pone.0191567.s007.doc]

**S7 Table. Summary of the redundancy analysis (RDA) of abundance of the most representative non-target arthropods collected by Pitfall traps in non-*Bt* (with and without insecticides) and *Bt* (DAS-81419-2) soybean fields at three sites over two to three years in Brazil.**

| Site (year) |  | Axes | | | | Total variance |
| --- | --- | --- | --- | --- | --- | --- |
|  |  | First | Second | Third | Fourth |  |
| Castro  (2012) | Eigenvalues | 0.061 | 0.028 | 0.016 | 0.012 | 1.000 |
|  | Species-environment correlations | 0.867 | 0.729 | 0.561 | 0.521 |  |
|  | Cumulative percentage variance |  |  |  |  |  |
|  | of species data | 13.1 | 19.2 | 22.6 | 25.3 |  |
|  | of species-environment relation | 44.8 | 65.5 | 77.1 | 86.1 |  |
|  | Sum of all eigenvalues |  |  |  |  | 0.463 |
|  | Sum of all canonical eigenvalues |  |  |  |  | 0.136 |
|  | Partition of variability (%) |  |  |  |  |  |
|  | Sampling time | 53.7 |  |  |  |  |
|  | Soybean treatments | 6.3 |  |  |  |  |
|  | Permutation test for canonical axes^1^ | F-ratio | *P*-value |  |  |  |
|  | First axis | 3.63 | 0.052 |  |  |  |
|  | All axes | 1.25 | 0.117 |  |  |  |
| Castro  (2013) | Eigenvalues | 0.030 | 0.018 | 0.012 | 0.011 | 1.000 |
|  | Species-environment correlations | 0.722 | 0.501 | 0.608 | 0.497 |  |
|  | Cumulative percentage variance |  |  |  |  |  |
|  | of species data | 6.2 | 10.0 | 12.6 | 14.9 |  |
|  | of species-environment relation | 29.8 | 48.4 | 60.5 | 71.8 |  |
|  | Sum of all eigenvalues |  |  |  |  | 0.477 |
|  | Sum of all canonical eigenvalues |  |  |  |  | 0.099 |
|  | Partition of variability (%) |  |  |  |  |  |
|  | Sampling time | 63.0 |  |  |  |  |
|  | Soybean treatments | 1.3 |  |  |  |  |
|  | Permutation test for canonical axes | F-ratio | *P*-value |  |  |  |
|  | First axis | 1.98 | 0.878 |  |  |  |
|  | All axes | 0.78 | 0.932 |  |  |  |
| Montividiu  (2011) | Eigenvalues | 0.079 | 0.060 | 0.032 | 0.021 | 1.000 |
|  | Species-environment correlations | 0.761 | 0.659 | 0.678 | 0.645 |  |
|  | Cumulative percentage variance |  |  |  |  |  |
|  | of species data | 12.4 | 21.9 | 27.0 | 30.3 |  |
|  | of species-environment relation | 36.4 | 64.2 | 79.2 | 88.7 |  |
|  | Sum of all eigenvalues |  |  |  |  | 0.633 |
|  | Sum of all canonical eigenvalues |  |  |  |  | 0.216 |
|  | Partition of variability (%) |  |  |  |  |  |
|  | Sampling time | 36.7 |  |  |  |  |
|  | Soybean treatments | 8.5 |  |  |  |  |
|  | Permutation test for canonical axes^1^ | F-ratio | *P*-value |  |  |  |
|  | First axis | 3.40 | 0.264 |  |  |  |
|  | All axes | 1.55 | **0.039** |  |  |  |
| Montividiu  (2012) | Eigenvalues | 0.114 | 0.039 | 0.032 | 0.025 | 1.000 |
|  | Species-environment correlations | 0.837 | 0.672 | 0.737 | 0.669 |  |
|  | Cumulative percentage variance |  |  |  |  |  |
|  | of species data | 19.4 | 26.0 | 31.4 | 35.6 |  |
|  | of species-environment relation | 47.9 | 64.2 | 77.4 | 87.8 |  |
|  | Sum of all eigenvalues |  |  |  |  | 0.589 |
|  | Sum of all canonical eigenvalues |  |  |  |  | 0.239 |
|  | Partition of variability (%) |  |  |  |  |  |
|  | Sampling time | 41.0 |  |  |  |  |
|  | Soybean treatments | 10.1 |  |  |  |  |
|  | Permutation test for canonical axes | F-ratio | *P*-value |  |  |  |
|  | First axis | 5.77 | **0.001** |  |  |  |
|  | All axes | 2.04 | **0.001** |  |  |  |
| Montividiu  (2013) | Eigenvalues | 0.048 | 0.037 | 0.023 | 0.014 | 1.000 |
|  | Species-environment correlations | 0.584 | 0.546 | 0.516 | 0.507 |  |
|  | Cumulative percentage variance |  |  |  |  |  |
|  | of species data | 9.0 | 16.0 | 20.3 | 22.9 |  |
|  | of species-environment relation | 36.8 | 65.4 | 82.8 | 93.5 |  |
|  | Sum of all eigenvalues |  |  |  |  | 0.535 |
|  | Sum of all canonical eigenvalues |  |  |  |  | 0.131 |
|  | Partition of variability (%) |  |  |  |  |  |
|  | Sampling time | 46.5 |  |  |  |  |
|  | Soybean treatments | 3.8 |  |  |  |  |
|  | Permutation test for canonical axes | F-ratio | *P*-value |  |  |  |
|  | First axis | 2.97 | 0.763 |  |  |  |
|  | All axes | 0.97 | 0.547 |  |  |  |
| Uberlândia  (2011) | Eigenvalues | 0.065 | 0.039 | 0.024 | 0.014 | 1.000 |
|  | Species-environment correlations | 0.816 | 0.736 | 0.692 | 0.557 |  |
|  | Cumulative percentage variance |  |  |  |  |  |
|  | of species data | 12.8 | 20.4 | 25.1 | 27.8 |  |
|  | of species-environment relation | 41.3 | 65.7 | 80.8 | 89.5 |  |
|  | Sum of all eigenvalues |  |  |  |  | 0.511 |
|  | Sum of all canonical eigenvalues |  |  |  |  | 0.159 |
|  | Partition of variability (%) |  |  |  |  |  |
|  | Sampling time | 48.9 |  |  |  |  |
|  | Soybean treatments | 5.0 |  |  |  |  |
|  | Permutation test for canonical axes^1^ | F-ratio | *P*-value |  |  |  |
|  | First axis | 3.52 | **0.025** |  |  |  |
|  | All axes | 1.35 | 0.055 |  |  |  |
| Uberlândia  (2012) | Eigenvalues | 0.151 | 0.059 | 0.025 | 0.014 | 1.000 |
|  | Species-environment correlations | 0.812 | 0.869 | 0.747 | 0.735 |  |
|  | Cumulative percentage variance |  |  |  |  |  |
|  | of species data | 24.0 | 33.3 | 37.4 | 39.6 |  |
|  | of species-environment relation | 55.8 | 77.5 | 86.8 | 92.0 |  |
|  | Sum of all eigenvalues |  |  |  |  | 0.630 |
|  | Sum of all canonical eigenvalues |  |  |  |  | 0.271 |
|  | Partition of variability (%) |  |  |  |  |  |
|  | Sampling time | 37.0 |  |  |  |  |
|  | Soybean treatments | 9.3 |  |  |  |  |
|  | Permutation test for canonical axes | F-ratio | *P*-value |  |  |  |
|  | First axis | 7.59 | 0.080 |  |  |  |
|  | All axes | 2.27 | **0.021** |  |  |  |

^1^Permutation test by 999 Monte Carlo permutations (α = 0.05).

*P*-values highlighted in bold are statistically significant.

The abundance of non-target arthropods was log(x + 1) transformed before analysis.
